# The clock gene BHLHE40 and atypical CCNG2 control androgen-induced cellular senescence as a novel tumor suppressive pathway in prostate cancer

**DOI:** 10.1186/s13046-024-03097-6

**Published:** 2024-06-20

**Authors:** Mehdi Heidari Horestani, Golnaz Atri Roozbahani, Aria Baniahmad

**Affiliations:** Institute of Human Genetics, Jena University Hospital, Am Klinikum 1, 07740 Jena, Germany

**Keywords:** Androgen receptor, Androgen-induced cellular senescence, Clock gene, Prostate cancer, Bipolar androgen therapy

## Abstract

**Background:**

The androgen receptor (AR) is a drug target used to inhibit AR and prostate cancer (PCa) growth. Surprisingly, treatment with supraphysiological androgen level (SAL), used in bipolar androgen therapy, inhibits growth of PCa suggesting a tumor-suppressive activity by SAL. SAL was shown to induce cellular senescence in PCa.

**Methods:**

RNA-seq and transcriptome analysis, ChIP-seq, human 3D PCa spheroids, mouse xenografted castration-resistant PCa, knockdown and overexpression, Co-immunoprecipitation (Co-IP), translocation analysis, immune detection, qRT-PCR, protein–protein interaction modelling.

**Results:**

Here, mice xenografts with castration-resistant PCa tumors show that SAL inhibits cancer growth in vivo suggesting that SAL activates a tumor-suppressive mechanism. RNA-seq and ChIP-seq revealed the clock gene BHLHE40 is a novel direct AR target. Compared to adjacent human prostate tissues, the expression of BHLHE40 is reduced in PCa tumors and associated with reduced survival. Knockdown suggests that BHLHE40 mediates SAL-induced cellular senescence including tumor spheroids. Interestingly, a large overlap of differentially expressed gene sets was identified between BHLHE40 and SAL leading to the identification of four classes of SAL-BHLHE40 transcriptome landscapes. Co-IP and modelling suggest binding of BHLHE40 to AR and their co-translocation into nucleus by SAL treatment. Further, RNA-seq and ChIP-seq analysis indicate that the atypical tumor suppressive cyclin G2 emerged as a novel downstream target of BHLHE40 and a mediator of SAL-induced cellular senescence.

**Conclusions:**

The data provide evidence of the tumor suppressive activity of SAL and a novel signaling by the AR-BHLHE40-CCNG2 axis for androgen-induced cellular senescence, linking circadian rhythm factor to androgen signaling as a novel tumor suppressive pathway.

**Supplementary Information:**

The online version contains supplementary material available at 10.1186/s13046-024-03097-6.

## Background

Prostate cancer (PCa) is a highly age-associated disease ranking among top cancer-related death in men [1]. The androgen receptor (AR) regulates the proliferation of PCa [2]. Initially, PCa is an androgen-sensitive tumor (CSPC), but it can evolve into a castration-resistant form (CRPC). AR-targeted therapy using androgen deprivation therapy in combination with AR antagonists [3] is commonly used to block AR activity to slow tumor growth. However, tumors often become resistant but the AR remains a critical factor for tumor proliferation also for CRPC [4].

Notably, there seems to be a paradox in how PCa cells respond to androgen levels. Treatment with supraphysiological androgen levels (SAL) also inhibits PCa growth, especially for cells that grow in low androgen environments [5]. This observation has led to clinical trials involving bipolar androgen therapy (BAT), which uses cycles of SAL combined with androgen deprivation currently in phase II TRANSFORMER and RESTORE trials for patients with CRPC [6–8]. SAL used in BAT is effective in inhibiting PCa cell growth, suggesting that SAL may induce a tumor-suppressive program [9]. We previously showed that SAL induces cellular senescence in adherent 2D and in 3D spheroids as well as in tissues derived from prostatectomies [10]. The SAL induced phosphorylation of AKT and activation of AKT signaling was shown to mediate in part SAL-induced cellular senescence in both human CSPC and CRPC tumor cell lines [5, 11].

Our transcriptome data of both C4-2 and LNCaP cell lines [11, 12] showed induction of BHLHE40 by SAL. BHLHE40, Basic Helix Loop Helix e40, also known as Differentially Expressed in Chondrocytes protein 1 (DEC1), has been shown as a biomarker of cellular senescence [13–15] and has been implicated in thyroid hormone-induced cellular senescence in PCa [16]. Interestingly, BHLHE40 was shown to act as a clock gene [17–19]. In the brain in the suprachiasmatic nuclei, the expression of BHLHE40 is under control of light [20]. However, the role of this clock gene in cancer is underinvestigated although changes of circadian rhythm has been associated with cancer [21].

However, the role of BHLHE40 in SAL-induced cellular senescence in prostate cancer is poorly understood. The functional role of BHLHE40 in SAL-induced cellular senescence in PCa by modulating BHLHE40 expression levels through knockdown (KD) and overexpression (OE) experiments in both CSPC LNCaP and the CRPC C4-2 cell lines were analyzed suggesting that BHLHE40 is part of SAL-induced cell senescence program. RNA-seq analysis of C4-2 BHLHE40 KD cells treated with or without SAL identified a large overlap of differentially expressed genes (DEGs) between SAL transcriptome and *BHLHE40* that could be clustered in four transcriptome landscapes. The atypical cyclin CCNG2 (Cyclin G2) acting as a tumor suppressor [22], is identified here as a novel downstream direct target gene of both BHLHE40 and AR by ChIP-seq and RNA-seq to mediate SAL-induced cellular senescence and linking clock genes with tumor suppression.

## Methods

### Cell culture and treatments

The LNCaP cell line, representing a castration-sensitive prostate cancer model, was acquired from Protopopov et al. in 2004 [23]. The C4-2 cell line, exhibiting castration-resistant characteristics in prostate cancer, was obtained from Thalmann et al. in 2007 [24]. As previously described, LNCaP and C4-2 cells were cultured in RPMI and DMEM media, respectively, at 37 °C in a humidified incubator with 5% CO2 [5]. 1nM R1881 (Merck; Germany; R0908), was defined previously serving as SAL [5], or 0.1% DMSO (ROTH; Germany; 4720.1) as a solvent control was used for cell treatments.

### Transfections of siRNA, shRNA-vector and overexpression of BHLHE40

To modulate BHLHE40 expression levels in order to investigate the role of BHLHE40 in cellular senescence induction by SAL, C4-2 and LNCaP cell lines were subjected to knockdown by shRNA, siRNA, or overexpression. For shBHLHE40 KD plasmid construction, the backbone EZ-Tet-pLKO-Puro plasmid was obtained from Addgene (#85,966). shRNA targeting BHLHE40 with the following sequence (5’GCCCTGCAGAGTGGTTTACAA3’) was inserted into the backbone. Scrambled plasmid was obtained from Addgene (#47,541) as a non-targeting control. For overexpression of BHLHE40, the cDNA sequence of BHLHE40 was purchased from Eurofins Genomics. pCDH-CMV-puro, was used to insert the BHLHE40 cDNA. The JetPrime reagent from PolyPlus Co (PolyPlus; France; 101,000,015), was utilized as per the manufacturer's instructions. Transfection experiments were conducted 48 h (h) before treating cells with SAL or DMSO. si-mediated knockdown was performed in both cell lines by using ON-TARGETplus Human *BHLHE40* siRNA with the ‘AAAGAGACGUGACCGGAUU’ sequence (Dharmacon; USA; L-010318–00-0010) with a final concentration of 25 nM. As a negative control, ON-TARGETplus nontargeting control siRNA with the ‘UGGUUUACAUGUUGUGUGA’ sequence (Dharmacon; USA; D-001810–04-20) was used. Moreover, for CCNG2 KD ON-TARGETplus Human *CCNG2* siRNA with the ‘CAUGAUGUGAUCCGGAUUA’ sequence was used (Dharmacon; USA; L-003217–00-0010). siRNAs were transfected by the DharmaFECT reagent (Dharmacon; USA; T-2003–02) according to the manufacturer’s protocol. Transfection was performed 24 h prior SAL or DMSO treatments.

### Senescence-associated *beta* galactosidase activity (SA-β-Gal) assays

For SA β-Gal activity, 50,000 cells for both C4-2 and LNCaP cell lines were seeded per well in a 6-well plate. The staining was performed as described previously [5]. Briefly, cells were washed with 1 × PBS prior fixation by 1% glutardialdehyde for 5 min 72 h post treatments. For the next step, cells were washed with 1 × PBS and incubated at 37 °C in SA β-gal staining solution. Staining solution contains 40 mM citric acid/sodium phosphate buffer (pH 6.0), 1 mg/ml X-gal (5-bromo-4-chloro-3-indolyl-β-D-galactopyranoside), 5 mM potassium ferrocyanide, 5 mM potassium ferricyanide, 150 mM sodium chloride, 2 mM magnesium chloride.

### Growth assays

Crystal violet was used to stain cells according to previously described protocol [25]. In short, 3 days after treatments, C4-2 and LNCaP cells were fixed with 1% glutaraldehyde and stained with 0.1% crystal violet to measure the cell population. Stained cells were washed with Sörenson’s solution which contains 0.9%, w/v tri-sodium citrate, 2% HCl, 40% ethanol and water. Absorbance was measured at 590 nm.

### mRNA isolation and qRT-PCR

RNA was isolated from the cells using RNA-solv reagent (omega; USA; R6830) according to the manufacture’s protocol. In brief, cell suspension was collected in a 1.5 ml Eppendorf tube. After adding RNA-solv and chloroform and through mixing, centrifugation was performed for phase separation. RNA was precipitated by isopropanol and centrifugation. RNA concentration was measured by Nanodrop ND-1000 Spectrophotometer. 2 µg RNA was converted to cDNA using a High-Capacity cDNA Reverse Transcription Kit (Applied Biosystems; Lithuania; 4,368,814), the SsoAdvanced Universal SYBR Green Supermix (Bio-Rad; USA; 1,725,271), gene specific primers, and Bio-Rad CFX Duet Real Time PCR machine. All primers used are listed in Supplemental Table S1. Plots represent the Mean of the expression levels and error bars represent SEM.

### Protein isolation and Western blot

Cells were lysed in 80 μl lysis buffer (20 mM Tris–HCl pH 8.0, 100 mM NaCl, 1 mM EDTA, 1% NP-40 and 1% Tergitol, 50 mM NaF, 100 μM Na_3_VO_4_, 10 mM β-Glycerophosphate) and centrifuged at 12 000 × g/4 °C for 10 min to obtain the cell extracts. Quantification of the protein concentration was performed with Nanodrop ND-1000 Spectrophotometer. Subsequently, 30 µg protein extract was loaded for Western blot and anti-beta Actin antibody served as loading control. Cell lysates were separated by 12% SDS-PAGE then proteins were transferred to PVDF membranes. Skim milk was used to block membranes then membranes were incubated with primary antibodies. The detection was performed by ImageQuantTM LAS 4000 (GE Healthcare Bio-Sciences AB). Quantification of bands was performed with the LabImage D1 software. All used antibodies are listed in Supplemental Table S2.

### Tumor spheroid generation, senescence activity staining and immunofluorescent assays

3D spheroids of C4-2 cells with and without BHLHE40 knockdown were generated according to previous protocol [10, 26]. In short, 1000 cells per well were seeded in 96-well ultralow attachment plates (PerkinElmer). The cells were centrifuged 3 times at 300 rpm for 3 min at room temperature (RT), plate was incubated at 37 °C, 5% CO_2_ to form a spheroid. After 24 h of seeding, spheroids were treated for 6 days with SAL or DMSO. Cell culture medium was refreshed every 3 days. On day 6, spheroids were fixed with 4% PFA for 20 min at RT following fixation, 3 times washing with 1 × PBS (pH 6) was performed. spheroids were incubated with SA-β-Gal staining solution overnight at 37 °C. Next day spheroids were washed with 1 × PBS, paraffin-embedded and dehydrated in Xylol for 10 min, 100% EtOH, 96% EtOH, 70% EtOH each for 5 min. All steps were repeated twice. Afterwards heat-induced antigen retrieval in a steamer was performed for 20 min in a 1 × citrate buffer. The slides were then washed with 1 × PBS and permeabilization was performed with 0.2% Triton X100 for 10 min. Slices were used for the imaging by brightfield microscope CellObserverZ1 (Carl Zeiss). For Ki-67 staining, spheroid slices were permeabilized and incubated for 1 h at RT in 5% normal goat serum (NGS) (Biozol; Germany; ENG9010-10) for blocking and incubated with primary anti-Ki67 antibody overnight. The next day, spheroids were washed 3 times with 1 × PBS each for 10 min then, secondary anti-rabbit IgG Alexa 546 antibody was used for 1 h at RT in the dark. Washing steps were performed, and DAPI (Life Technologies; USA; H3569) solution (1 μg/ml in 1 × PBS) was used for nuclei staining for 10 min. After one time washing with 1 × PBS for 10 min, the spheroid slices were covered with Fluoromount-G® (SouthernBiotech; USA; 0100–01) and coverslips. Images were captured with the confocal laser scanning microscope (Carl Zeiss LSM 880). For immunofluorescence of BHLHE40, 15,000 LNCaP and C4-2 cells were seeded in Chambered Borosilicate Cover glass from Lab-Tek. Next day cells were treated with SAL or DMSO for 3 days. After seeding for 3 days. After 3 times washing cells with 1 × PBS and permeabilization with 0.2% Triton X100 for 10 min, cells were washed again with 1 × PBS and same as spheroid slices, cells were incubated 1 h at RT with NGS. All following steps were according to steps mentioned above. Antibodies are listed in Supplemental Table S2.

### Ex vivo prostatectomy sample treatment

The ex vivo treatment of PCa samples from patients with radical prostatectomies was described previously with ethical approval (Reg.-Nr.: 2019–1502) [11, 27]. All the patients gave informed consent, and all were informed about the purpose of the study and conform to the Declaration of Helsinki. One prostatectomy sample was used from each patient. samples were treated for 48 h by DMSO or R1881. RNA was extracted same as the protocol mentioned in the RNA isolation section. For RT-qPCR, two technical replicates for each sample were performed. Expression levels were normalized to housekeeping genes, alpha-Tubulin and TBP. Sequence of the primers are mentioned in Supplemental Table S1. Gleason scores of prostatectomy samples are listed in Table S3.

### Mouse xenograft experiments

Animal experiments were approved by the Thüringer Landesamt für Lebensmittelsicherheit und Verbraucherschutz, Germany (Reg.-Nr.: UKJ-23–013). C4-2 cell suspension (10^6^ cells per 50 μl 1 × PBS) was mixed 1:1 with Matrigel (CORNING; USA; 356,231) and injected subcutaneously (s.c.) into both flanks of the intact (non-castrated) nude mice (8-week-old male athymic nude mice, Janvier Labs, France). After the tumors reached a size of approximately 80 mm^3^, vehicle (0.5% Tween 80) or dihydrotestosterone (DHT, 50 mg/kg) corresponding SAL (AbMol BioScience; USA; M6033) was s.c injected daily. Tumor size was measured every 48 h using a caliper (tumor volume = (length × width^2^) × 0.52). Two-way ANOVA was used for statistical analysis. Mice were weighted every other day and sacrificed when tumor size reached a size of approximately 800 mm^3^ or if weight loss exceeded 20% of initial weight or injection reached to 5 weeks. Tumors were frozen in liquid nitrogen and RNA was extracted from tumors same as previously mentioned protocol [11].

### RNA-Seq and data analysis

For transcriptome analyses total RNA was isolated from C4-2 cells with and without BHLHE40 KD cells treated with and without SAL treatment, for 72 h in three independent biological replicates, using the mentioned protocol in section mRNA isolation and qRT-PCR. Samples were sent for paired-end sequencing to macrogen Europe. Samples quality check was performed using FastQC version 0.11.5. Adapters were trimmed from both ends by using Skewer version 0.2.1 [28] in Linux (Ubuntu). hg38 (BSgenome.Hsapiens.UCSC.hg38) as a reference genome was used for alignment. Alignment was performed by using the QuasR package and using Rhisat2 aligner algorithm. Read counting was carried out by using GenomicAlignments package. Normalization was performed by using DESeq2 package. Two replicates with confirmed regulation of known BHLHE40 target genes by knockdown were used for further analysis. GSEA (Gene set enrichment analysis) was performed using GSEA (RRID:SCR_003199) [29]. Pathway analysis was performed by using PathFindR and ClusterProfiler packages [30–32]. The motif analysis for ChIP-seq data was performed via HOMER (RRID:SCR_010881).

AR ChIP-Seq was analyzed for finding AR binding sites under DHT treatment corresponding to SAL and overlapping genes between AR and RNA-Seq data from BHLHE40 Knockdown cells [33]. BHLHE40 ChIP-Seq was used for analysis to identify genome-wide BHLHE40 chromatin binding sites [34]. Two RNA-Seq datasets were used to analyze the expression level of BHLHE40 in prostatectomy samples compared to adjacent tumors [35, 36].

### Prediction of transcription factor binding site and identifying gene network connection

The promoter sequence of target gene was downloaded from UCSC Genome Browser (RRID:SCR_005780) [37] and the JASPAR (RRID:SCR_003030) [38] was used to predict binding sites of BHLHE40 in the promoter of target gene. The cytoHubba (RRID:SCR_017677) from Cytoscape (RRID:SCR_003032), and the STRING (RRID:SCR_005223) [39] were utilized to explore the established network connections between BHLHE40 and CCNG2.

### Co-immunoprecipitation assays

Co-immunoprecipitation (Co-IP) experiments were performed as described previously [40]. Briefly, a specific antibody against BHLHE40 or normal rabbit IgG as a negative control was incubated with protein A magnetic beads. After extensive washing of beads, cell extracts from C4-2 and LNCaP cells were incubated with the antibody-loaded beads for 2 h at 4 °C. After washing steps, beads were resuspended in SDS buffer and boiled at 99 °C. SDS buffer containing precipitated protein complex was loaded to 12% SDS-PAGE for detection by Western blotting. Antibodies used with concentrations are listed in Supplemental Table S2.

### Protein–protein interaction modeling

Protein structure prediction for BHLHE40 and AR ligand binding domain with hinge region were performed by I-TASSER (RRID:SCR_014627) [41]. The C-score, a measure of confidence in the model, ranged from [-5,2]. A higher score indicates a higher confidence in the model. Also, the TM score > 0.5 indicates correct topology, while TM < 0.17 represents random similarity in the predicted model [42–44]. Also, protein structure of AR-LBD domain with SAL was downloaded from RCSB Protein Data Bank (PDB ID 1E3G) as a positive control. Docking was performed via PatchDock (RRID:SCR_017589) [45] and ClusPro (RRID:SCR_018248) [46]. Visualization was performed in PyMOL version 1.8.0.0 (RRID:SCR_000305).

### Statistical analysis

The two-tailed student t-test was used for the comparison of the mean values between two groups and two-way ANOVA was used for multiple comparisons in the GraphPad Prism version 8.0 (RRID:SCR_000306).

All statistics represent the Mean of values and error bars represent SEM. For multiple comparisons, the post-hoc (Tukey) test was used.

## Results

### SAL induces cellular senescence and BHLHE40 KD reduces it

**Fig. 1:**
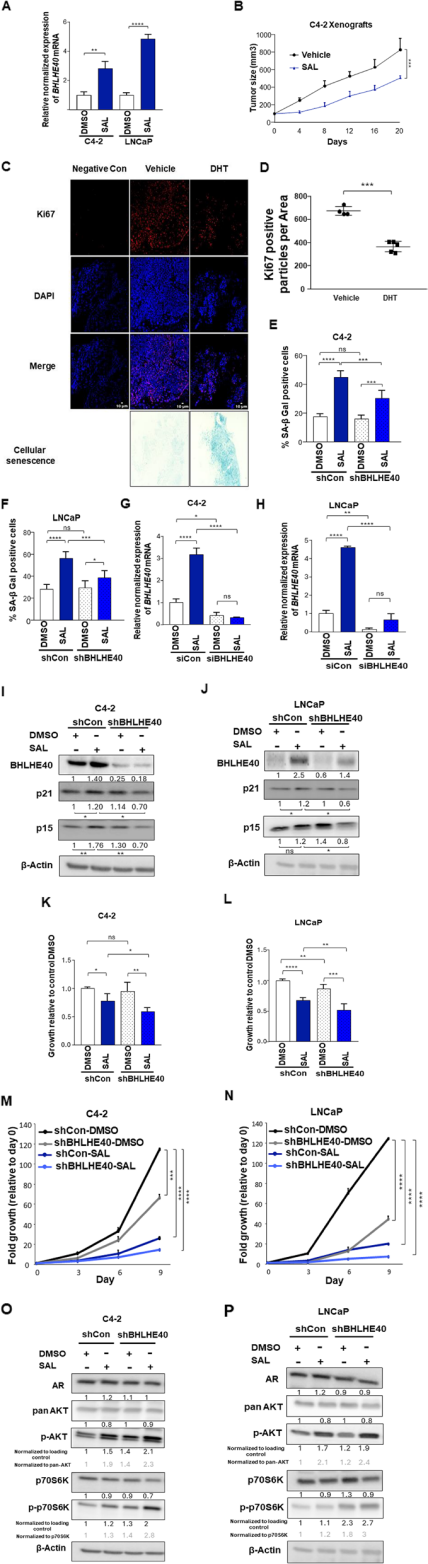
The knockdown of BHLHE40 reduces SAL-induced cellular senescence. **A** qRT-PCR of SAL treated LNCaP and C4-2 cells detecting the change of expression by SAL (*n* = 3) normalized to two house-keeping genes (α-Tubulin and TBP). SAL induces *BHLHE40* in both cell lines. **B** Tumor size analysis of C4-2 xenografted mice reveals reduction in the tumor growth by SAL using DHT (50 mg/kg) (*n* = 5) compared to vehicle (*n* = 4) using two-way ANOVA. **C **Depicting the Ki67 positive cells and SA β-Gal activity. IF staining applied to detect the Ki67 positive cells in C4-2 mice-xenografted tumors. In SAL-treated tumors, the number of positive cells is reduced. Samples without anti-Ki67 serve as a negative control. Also, senescence level is induced by SAL (*n* = 5) compared to vehicle (*n* = 4). **D** Quantification of Ki67 positive cells per area was measured by using ImageJ software. **E** and** F** BHLHE40 KD was performed in both cell lines and subsequently treated with SAL 72 h prior the SA β-Gal activity detection (*n* = 3) SAL induces cellular senescence and BHLHE40 KD reduces it. **G** and **H **qRT-PCR of BHLHE40 KD cells treated with SAL or DMSO. Knockdown significantly reduces *BHLHE40* in both cell lines. **I** and** J **Western blots of extracts from treated cells (*n* = 3) detecting changes of p15^INK4b^ and p21^WAF1/Cip1^ by SAL and BHLHE40 KD. β-Actin serves as the loading control. Numbers below the bands indicate changes in intensities normalized to that of β-Actin. **K** and **L** Crystal violet staining after 72 h treatment analyzing growth of SAL-treated and BHLHE40 KD in both cell lines. **M** and **N** Growth curve analyzing growth of SAL-treated and BHLHE40 KD in both cell lines with day 0 set arbitrarily as 1 (*n* = 3). **O** and **P **Western blot of treated cells with SAL and BHLHE40 KD. AR is not affected by BHLHE40 KD. Phosphorylation of AKT and p70S6K are induced by BHLHE40 KD and SAL. The ratio of p-AKT versus AKT and p70S6K versus p-p70S6K are indicated in grey. *P* value < 0.0001 = ****, 0.001 = ***, < 0.01 = **, < 0.05 = *, ns = non-significant

Transcriptome analyses of SAL treated PCa cells [11, 12] revealed induction of *BHLHE40* mRNA encoding BHLHE40/DEC1. The induction of *BHLHE40* was confirmed by qRT-PCR in both LNCaP and C4-2 cell lines treated with SAL or DMSO as the solvent control (Fig. 1A). AR ChIP-Seq data from DHT treated C4-2 cells at SAL suggests a direct binding of AR to the downstream of the *BHLHE40* gene (Supplemental Fig. S1A). Sequence analysis of the peak revealed the existence of several binding sites for ARE and ARE half-sites [47] within this peak suggesting that *BHLHE40* gene is a novel direct AR upregulated target gene by androgen. Since SAL induces cellular senescence in both C4-2 and LNCaP PCa cell lines [5], induction of senescence and growth inhibition were analyzed in C4-2 xenografted mice model treated with SAL or vehicle. Measuring the tumor sizes reveals significant reduction of tumor growth (Fig. 1B). Analyzing the Ki67 as a proliferation marker confirmed the reduction of growth by a smaller number of positive cells for Ki67 in SAL treated tumors in compared to vehicle (Fig. 1C and D). Moreover, analyzing the senescence associated beta-galactosidase activity (SA b-Gal) by brightfield microscopy imaging suggests induction of cellular senescence in SAL treated tumors compared to vehicle (Fig. 1C) suggesting that the in vivo results confirm the inhibitory effect of SAL on growth and its induction of cellular senescence in vitro [11]. To analyze whether BHLHE40 mediates SAL-induced cellular senescence knockdown (KD) experiments were performed in both C4-2 and LNCaP cell lines. The SA b-Gal activity results suggest that SAL-induced senescence levels were significantly reduced in BHLHE40 KD of both cell lines (Fig. 1E, F, S1B, S1C) indicating that BHLHE40 mediates in part cellular senescence in both PCa cell lines induced by SAL. The BHLHE40 KD was verified at mRNA and protein levels (Fig. 1G-J ) and by induced expression of *BHLHE41* mRNA, which is a BHLHE40 repressed direct target gene [48] (Fig. S1G, S1H) confirming a functional KD of BHLHE40. Accordingly, the senescence marker p15^INK4b^ is increased by SAL and decreased by KD of BHLHE40 under SAL treatment. The cell cycle inhibitor p21^WAF1/Cip1^ which also consider as a cellular senescence marker [49], was induced slightly by SAL, which is in line with previous findings [5] and reduced by BHLHE40 KD in both cell lines (Fig. 1I, J). These findings collectively indicate that SAL induces cellular senescence, which is in part mediated through BHLHE40. Intriguingly, BHLHE40 KD reduces the SAL-inhibited cell growth in both cell lines and also in control-treated LNCaP cells suggesting that BHLHE40 regulates cell proliferation by another pathway in addition to cellular senescence (Fig. 1K-N) without signs of apoptosis (Fig. S2A, S2B). These findings indicate that BHLHE40 regulates growth and cellular senescence induced by SAL in both cell lines and suggests a novel AR- BHLHE40 axis to control androgen-induced cellular senescence.

### BHLHE40 KD induces phosphorylation of AKT and p70S6K

The AKT pathway has been identified as a critical pathway in controlling pro-survival signaling SAL-induced cellular senescence in PCa and it was observed that SAL enhances phosphorylation of AKT at serine-473 [11]. Interestingly however, the KD of BHLHE40 rather further enhances p-AKT levels and p-p70S6K as a downstream factor of AKT although cellular senescence levels are decreased by the KD (Fig. 1O, P) suggesting that BHLHE40 rather inhibits the AKT signaling, which may be one tumor suppressive pathway mediated by BHLHE40. Thus, these findings suggest that BHLHE40 inhibits AKT phosphorylation and its downstream signaling in PCa cells.

The KD of BHLHE40 does not seem to modulate AR protein level and the expression of AR target genes (Fig. 1O, P, Fig. S1D-F). This indicates that BHLHE40 may only weakly regulate AR transcriptional signaling. Our previous RNA-seq data, using the AKT inhibitor AKTi, indicate a slight induction of *BHLHE40* in C4-2 cell (Fig. S2C, S2D) [11] indicating that AKT signaling inhibits BHLHE40 expression likely in an indirect manner as a potential feed-back loop.

Collectively, the obtained data suggest in addition to the well-established p-AKT signaling, the existence of another signaling pathway that mediates SAL-induced cellular senescence by BHLHE40 indicating an alternative AKT pathway for SAL-induced cellular senescence.

### BHLHE40 KD reduces cellular senescence of multicellular CRPC 3D tumor spheroid model

**Fig. 2:**
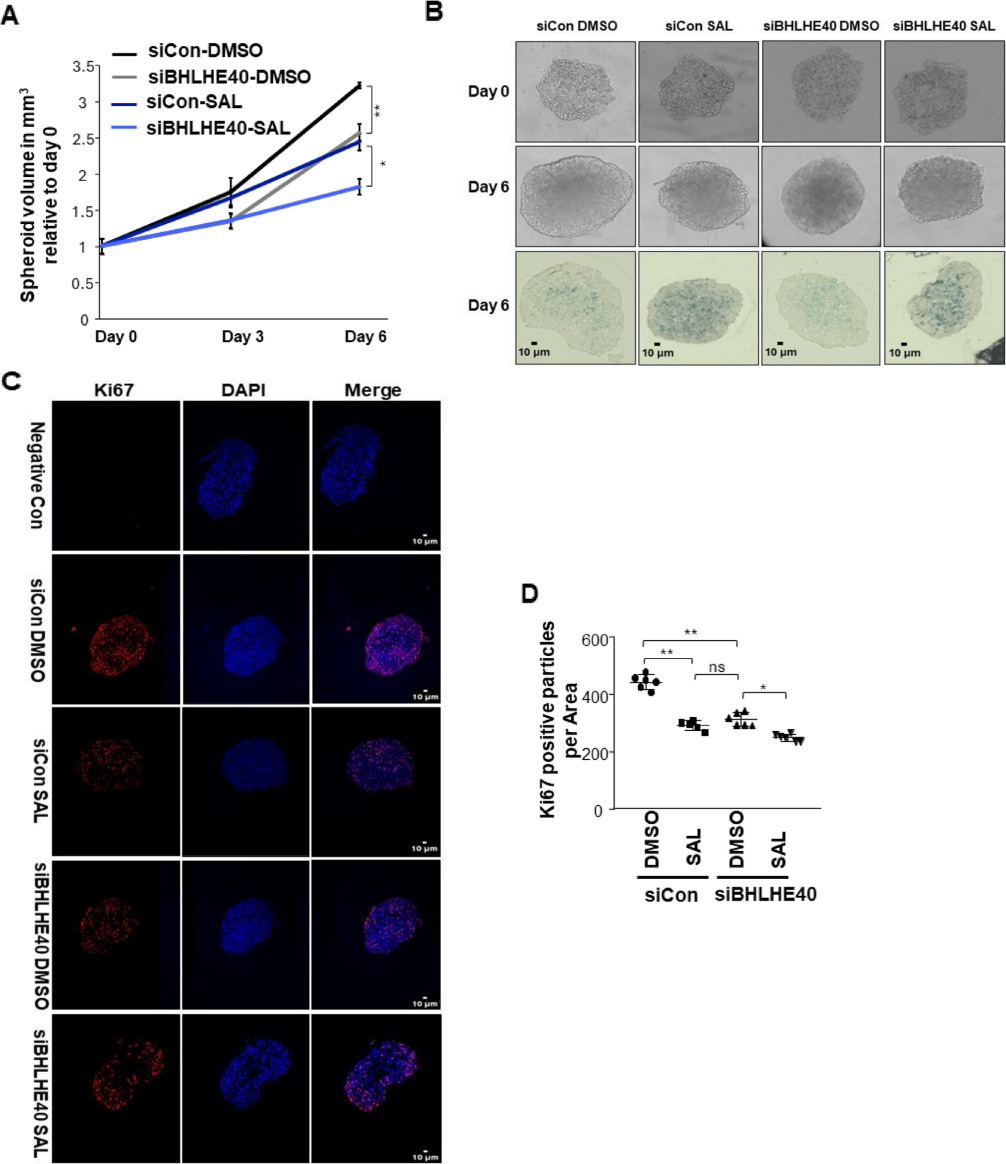
BHLHE40 KD reduces cellular senescence and Ki67 growth marker in 3D tumor spheroid model. **A** Tumor volume analysis in C4-2 cell line revealed reduction in the growth of tumor spheroids by SAL and further reduction by BHLHE40 KD (*n* = 2 independent biological replicates with each 10 technical replicates). **B** Depicting the size of tumor spheroids and activity of senescence-associated beta-galactosidase (SA β-Gal). Senescence level is induced by SAL treatment and reduced after BHLHE40 KD (*n* = 2). **C **Immunofluorescence staining applied to detect the Ki67 positive cells in spheroids. In SAL treated spheroids, the number of positive cells for Ki67 is reduced and more reduction is detected by BHLHE40 KD. Samples without primary anti-Ki67 antibody serve as a negative control (*n* = 2). **D **Quantification of Ki67 positive cells was measured by using ImageJ software. *P* value < 0.01 = **, < 0.05 = *, ns = non-significant

The 3D tumor spheroid model system is considered to reflect better the complexity of a tumor compared to 2D adherent culture [50]. 3D spheroids were generated from parental C4-2 and BHLHE40 KD cells using ultra-low attachment plates as described previously [12]. Confirming the findings from the 2D cultures, SAL reduces spheroid volumes (Fig. 2A). The BHLHE40 KD resulted in a further reduction of spheroid size in SAL-treated samples (Fig. 2A, B). SA b-Gal activity of the tumor spheroid slices shows an increase in staining by SAL that is reversed in the BHLHE40 KD spheroids (Fig. 2B). Immunofluorescence of Ki67, as a marker of proliferation suggests more Ki67 stained cells in control treated spheroid slices compared to SAL-treated ones, which is consistent with the higher levels of senescent cells observed in SAL treated samples (Fig. 2C, D). ImageJ software was used for quantification of Ki67 images. BHLHE40 KD spheroids resulted in fewer Ki67-positive cells compared to control DMSO samples, with a further reduction observed under SAL treatment in BHLHE40 KD samples (Fig. 2C, D). These findings are in accordance with the results from the 2D adherent cultures providing further evidence that BHLHE40 KD reduces cellular senescence in PCa cells also in 3D spheroids without enhancing growth. The lack of growth induction may be explained by the involvement of BHLHE40 in other cellular mechanisms, such as different types of cellular dormancy [51].

### Ex vivo SAL-treated prostatectomy samples and SAL-treated-CRPC mouse xenografts induce the expression of *BHLHE40*

**Fig. 3:**
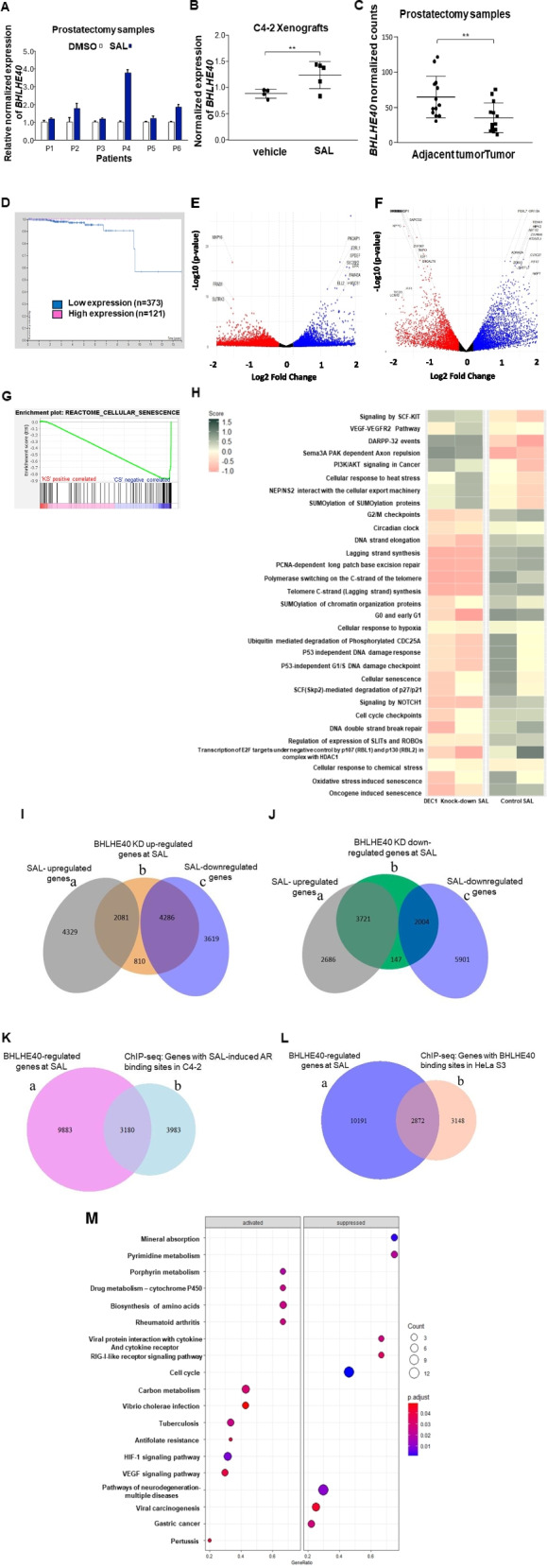
SAL induces *BHLHE40* expression and *BHLHE40* RNA-seq shows strong overlap to AR and BHLHE40 ChIP-seq. **A** qRT-PCR of *ex-vivo* SAL-treated prostatectomy samples shows higher expression of *BHLHE40* compared to paired DMSO. **B** *BHLHE40* mRNA was analyzed with qRT-PCR in C4-2 mice xenografts treated with SAL. Significant induction of *BHLHE40* is measured in tumors treated with SAL (*n* = 5) compared to the vehicle (*n* = 4). **C** RNA-Seq data from prostatectomy patients compared to adjacent tumor revealed low level of *BHLHE40* in tumors. **D** Kaplan Meier survival plot showed low level of BHLHE40 is associated with the less survival in prostate cancer. **E** Volcano plot shows log2FC of samples treated with SAL vs. DMSO. **F** Volcano plot of BHLHE40 KD at SAL vs. control at SAL. **G **GSEA analysis depicts negative association of BHLHE40 KD at SAL with cellular senescence pathway compared to control samples at SAL (‘KS’ is BHLHE40 KD at SAL and ‘CS’ is control at SAL). **H** Heatmap plot shows the direction of pathways between BHLHE40 KD at SAL and SAL-treated control (log2FC). **I** and **J** Transcriptome analysis of DEGs and overlaps between up- and downregulated genes by SAL and BHLHE40 at SAL. **I** Venn diagram depicts overlap between SAL treated genes and BHLHE40 KD up-regulated genes at SAL. a. SAL up-regulated, b. BHLHE40 KD induced genes at SAL c. SAL down-regulated. **J** a. SAL up-regulated, b. Suppressed genes by BHLHE40 KD at SAL c. SAL down-regulated. **K** Venn diagram indicates overlap genes between BHLHE40 KD at SAL samples with AR ChIP-seq in C4-2 cells. **L** Venn diagram depicts common genes between BHLHE40 KD at SAL with BHLHE40 ChIP-seq data in Hela-S3 cell line. **M** Pathway analysis performed by R Package for BHLHE40 KD RNA-Seq and BHLHE40 ChIP-seq overlap genes. *P* value < 0.01 = **

To analyze the androgen-controlled expression of *BHLHE40* in native tissues, samples from radical prostatectomy were subjected to ex vivo treatment with either DMSO or SAL for two days. The results revealed an increase in the BHLHE*40* mRNA in some of the samples treated with SAL (Table S3 and Fig. 3A). Moreover, *BHLHE40* mRNA was investigated in C4-2 xenograft mice model treated with either SAL or vehicle. Data show induction of *BHLHE40* mRNA in the tumors of SAL-treated mice (Fig. 3B). In addition, *BHLHE40* expression was compared between tumor and adjacent samples derived from two distinct GEO datasets. *BHLHE40* mRNA is significantly diminished in PCa samples compared to adjacent tumor tissues (Fig. 3C) supporting the notion that BHLHE40 has tumor suppressor function. These findings are further corroborated by the Kaplan–Meier survival plot for BHLHE40 in PCa derived from the TCGA database with low *BHLHE40* expression linked to reduced survival (Fig. 3D). Collectively, these data propose that BHLHE40 is upregulated by SAL in 2D, 3D spheroids, ex vivo and in vivo, and that BHLHE40 mediates androgen-induced cellular senescence, which serves as a tumor suppressor pathway in PCa.

### Transcriptome landscape of BHLHE40 and pathway analysis indicates a large overlap to androgen transcriptome

Volcano plots of RNA-seq analyses of C4-2 BHLHE40 KD cells treated with DMSO or SAL visualize the overall fold change in gene expression levels by SAL-treated and the BHLHE40 KD (Fig. 3E, F, and Fig. S3). Gene Set Enrichment Analysis (GSEA) of SAL-treated BHLHE40 KD samples versus their related control show a negative association of BHLHE40 KD with the cellular senescence pathway confirming a positive regulation of cellular senescence by BHLHE40 (Fig. 3G). Pathway analyses uncovered several interesting pathways related to cellular senescence, cell cycle regulation, circadian clock, DNA repair, and PI3K/AKT signaling in cancer (Fig. 3H and Table 1). Comparing the expression levels of genes in BHLHE40 KD SAL-treated samples with those of their paired controls revealed a reduction in the ‘cellular senescence’, an induction in the ‘PI3K/AKT signaling in cancer’, and a reduction in the ‘circadian clock’ pathways (Fig. 3H).

**Table 1:** Pathway-log2FC of KD BHLHE40 SAL vs. Control SAL

	*p*-value
Regulation of expression of SLITs and ROBOs	4.78E-28
Signaling by ROBO receptors	3.84E-27
RHOA GTPase cycle	1.67E-18
VEGFA-VEGFR2 Pathway	4.78E-05
Signaling by WNT	4.87E-05
p53-Dependent G1 DNA Damage Response	0.000218
DNA Repair	3.89E-19
**Cellular Senescence**	2.93E-05
Signaling by NOTCH1 in Cancer	0.000195
**Oncogene Induced Senescence**	3.95E-06

Given that SAL induces the expression of BHLHE40 and that the BHLHE40 KD reduces the senescence level in PCa cell lines, transcriptome data were analyzed and classified into subsets of genes dependent on their up- or downregulation to identify specific AR-BHLHE40 transcriptome landscapes and specific regulated pathways by these two transcription factors. To achieve this, upregulated DEGs from the BHLHE40 KD SAL-treated samples were separated from the downregulated and each set was separately analyzed for their overlap with the DEGs of SAL-treatment. The Venn diagram depicts approximately 6300 differentially upregulated genes in BHLHE40 KD at SAL that were common with DEGs in SAL-induced samples. Only about 800 genes were upregulated, being unique to BHLHE40 KD SAL-treated samples (Fig. 3I). This suggests a large overlap of genes that are regulated by BHLHE40 and are co-regulated by SAL. The same analysis was performed for the downregulated gene set. Similarly, a large number of DEGs were common but only nearly 140 genes were downregulated and specific to BHLHE40 (Fig. 3J). Taking together, we identified four sets of genes being co-regulated by BHLHE40 and AR.

Pathway analysis was performed for common genes between BHLHE40 KD up-regulated and SAL down-regulated from Fig. 3I. Also, for BHLHE40 KD down-regulated and SAL up-regulated overlap genes from Fig. 3J. Notably, several pathways associated with cellular senescence emerged (Tables 2 and 3).

**Table 2:** Pathways-log2FC of common genes between “b” and “c” in Fig. 3 I

	*p*-value
Signaling by WNT	2.09E-09
PIP3 activates AKT signaling	5.05E-09
Negative regulation of the PI3K/AKT network	4.30E-08
Mitotic G2-G2/M phases	5.36E-07
Cell cycle checkpoint	4.03E-06
**Circadian Clock**	6.27E-06
PI3K/AKT Signaling in Cancer	9.56E-06
Senescence-Associated Secretory Phenotype (SASP)	0.017897
**FOXO-mediated transcription**	0.002727

**Table 3:** Pathways-log2FC of common genes between “a” and “b” in Fig. 3 J

	*p*-value
DNA Double Strand Break Response	3.20E-12
Recruitment and ATM-mediated phosphorylation of repair and signaling proteins at DNA double strand breaks	2.48E-12
Processing of DNA double-strand break ends	1.55E-10
**DNA Repair**	5.11E-11
**Oncogene Induced Senescence**	9.18E-06
**DNA Damage/Telomere Stress Induced Senescence**	0.000214
**Cellular senescence**	1.30E-11
G2/M Checkpoints	1.44E-11
**FOXO-mediated transcription**	2.83E-06

The FOXO-mediated transcription pathway is noteworthy as it appeared in both sets of common factors, albeit with different gene sets. This pathway is linked to cell cycle arrest, quiescence, growth, and stress resistance of cells [52–54]. In addition, specific sets of genes for BHLHE40 KD from Fig. 3I and J were analyzed for pathways. Most of the known BHLHE40-involved pathways were identified, such as the circadian clock and epithelial-mesenchymal transition (Fig. S4, Table S4).

Based on the large DEG overlap at SAL between BHLHE40 and AR we analyzed the coregulation of BHLHE40 with direct AR target genes. The overlap of genes from publicly available AR ChIP-seq data of SAL-treated C4-2 cells with our transcriptome obtained by SAL-treated BHLHE40 KD samples was examined (Fig. 3K). It emerged specific pathways such as cellular senescence as well as the FOXO-mediated transcription pathway (Table 4 and Fig. S5A, B).

**Table 4:** Pathways-common genes between ChIP-seq of AR with BHLHE40 KD SAL-treated RNA-seq

	*p*-value
PI3K/AKT Signaling in Cancer	3.50E-15
DNA Repair	2.66E-14
**FOXO-mediated transcription**	5.09E-13
Signaling by VEGF	4.76E-12
Circadian Clock	8.64E-12
Signaling by WNT	4.57E-09
Signaling by NOTCH	1.90E-08
Oncogenic MAPK signaling	3.83E-08
**Cellular Senescence**	1.99E-07
**Senescence-Associated Secretory Phenotype (SASP)**	0.002897

**Table 5:** Pathways-common genes between ChIP-seq of BHLHE40 with BHLHE40 KD SAL-treated RNA-seq

	*p*-value
RHO GTPase cycle	2.93E-11
Signaling by ROBO receptors	8.18E-06
Regulation of expression of SLITs and ROBOs	1.65E-05
VEGFA-VEGFR2 Pathway	0.000101
Transcriptional regulation by RUNX2	0.000453
Signaling by WNT	0.000984
**FOXO-mediated transcription**	4.20E-07
p53-Dependent G1 DNA Damage Response	0.00703
DNA Repair	0.014108

**Fig. 4:**
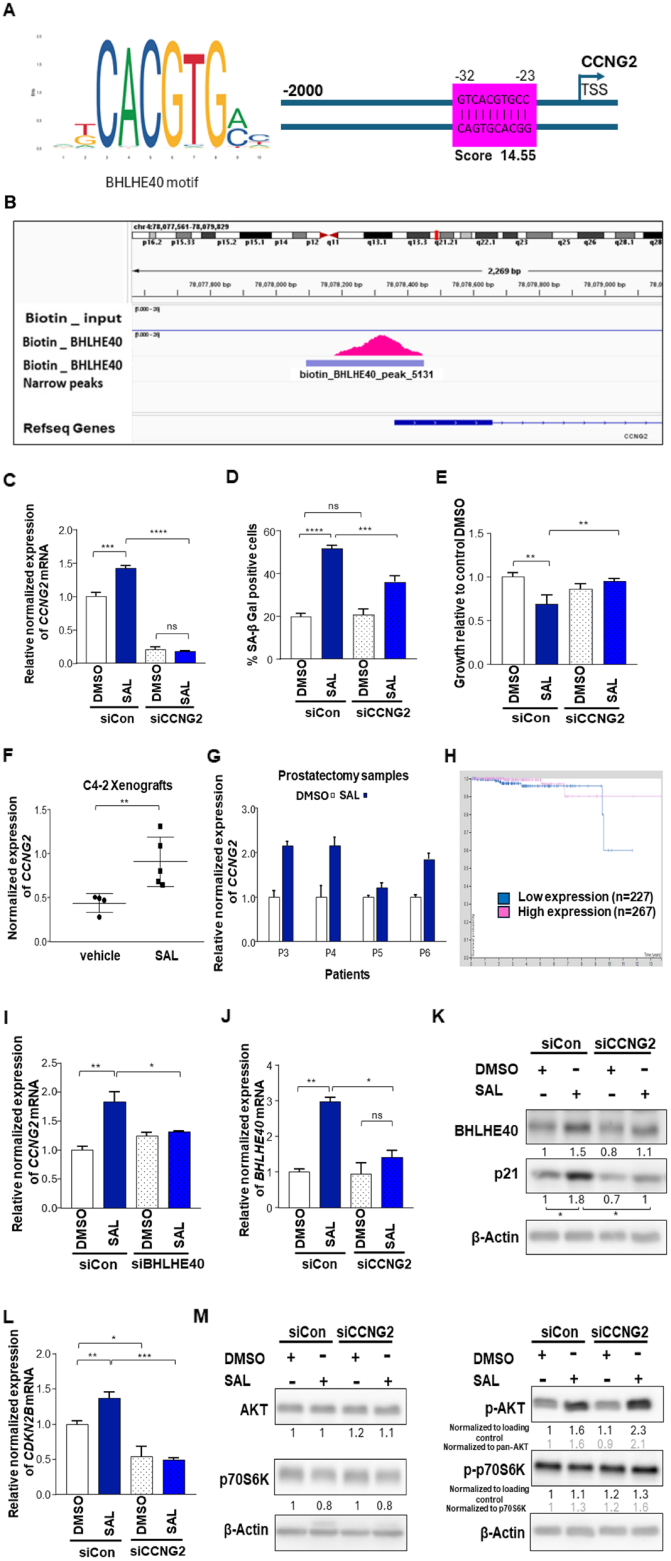
SAL induces *CCNG2* expression and BHLHE40 directly binds to CCNG2 gene and mediates cellular senescence. **A** Motif binding site prediction analysis depicts BHLHE40 binding in the close promoter region of *CCNG2* genes. **B** IGV software was used to visualize BHLHE40 binding site in the promoter of *CCNG2*. BHLHE40 ChIP-seq confirmed motif binding site prediction analysis. **C** Efficiency of CCNG2 KD evaluated by qRT-PCR (*n* = 3). **D** SAL induces cellular senescence and KD of CCNG2 reduces that induction in C4-2 cell line (*n* = 3). **E** crystal violet staining to analyze growth of SAL treated and CCNG2 KD in C4-2 cell line (*n* = 3). **F** qRT-PCR of C4-2 tumor xenografts from mice treated with SAL (*n* = 5) indicates significant induction of *CCNG2* mRNA compared to vehicle (*n* = 4). **G** qRT-PCR of SAL e*x-vivo* treated prostatectomy samples reveals induction in the expression level of *CCNG2*. **H** Kaplan Meier survival plot showed less survival with low level of CCNG2 in prostate cancer.** I** BHLHE40 KD reduces mRNA level of *CCNG2* in C4-2 cell line revealed by qRT-PCR (*n* = 3).** J** *BHLHE40* mRNA is reduced by CCNG2 KD in C4-2 cell line (*n* = 3). **K** CCNG2 KD reduces BHLHE40 protein, also SAL induction of p21^WAF1/Cip1^ is reduced by CCNG2 KD in C4-2 cells (*n* = 3). **L** mRNA level of *CDKN2B,* as a marker of senescence, is reduced by CCNG2 KD in C4-2 cells measured by qRT-PCR. **M** Changes of AKT and p70S6K phosphorylation by CCNG2 KD. The ratio of p-AKT versus AKT and p70S6K versus p-p70S6K are indicated in grey. *P* value 0.001 = ***, < 0.01 = **, < 0.05 = *

Given that the FOXO-mediated transcription pathway was identified in many subsets of the analysis, Cyclin G2 (CCNG2), a salient gene in this pathway was selected for further analysis. In contrast to other cyclins, CCNG2 levels are higher in cycle-arrested cells and mediates growth inhibitory in cancer [22, 55, 56]. Bioinformatic analysis predicts that BHLHE40 has a high score binding site in the close promoter region of the CCNG2 gene (Fig. 4A, S6A). BHLHE40 ChIP-seq data from HeLa cells was obtained from GEO to validate this prediction. The analysis suggests a specific binding site that aligns perfectly with the prediction model for BHLHE40 within the promoter region of the CCNG2 gene (Fig. 4B). Interestingly, BHLHE40 is recruited to its own gene. Moreover, AR ChIP-seq data from SAL treated C4-2 cells suggests a direct binding of AR to the upstream of the *CCNG2* gene (Fig. S6B) suggesting that *CCNG2* gene is also a novel direct AR upregulated target gene and transcriptionally co-regulated by BHLHE40.

Furthermore, pathway analysis of common genes between available BHLHE40 ChIP-seq data and our BHLHE40 KD SAL-treated transcriptome (Fig. 3L) yielded cell cycle regulation, DNA repair, and interestingly the FOXO-mediated transcription pathway (Fig. 3M and Table 5).

Moreover, the cytoHubba from Cytoscape, and the STRING were utilized to explore the established network connections between BHLHE40 and CCNG2, along with several cellular senescence markers (Fig. S7) [57–59]. The data suggest that BHLHE40 and CCNG2 are connected to each other in multiple ways and also to factors that regulate chromatin organization and cell cycle. Notably, the analysis indicates close interaction with cellular senescence markers and the potent cell cycle inhibitors p15^INK4b^, p16^INK4a^, and p21^WAF1/Cip1^.

### CCNG2 KD decreases cellular senescence under the influence of SAL treatment

Based on the bioinformatic predictions and the regulation of CCNG2 by BHLHE40 and AR, the hypothesis was that CCNG2 is part of the SAL-induced cellular senescence. Knockdown of *CCNG2* by siRNA indicates that SAL enhances *CCNG2* mRNA expression (Fig. 4C) and reduces the SAL-induced cellular senescence (Fig. 4D, S8A). This suggests that CCNG2 mediates SAL-induced cellular senescence being in line with enhanced growth for CCNG2 KD-treated SAL samples (Fig. 4E, S8B). Also, in the C4-2 xenograft tumors and ex vivo treated prostatectomy samples the expression level of *CCNG2* was induced by SAL (Fig. 4F, G). Further, the Kaplan–Meier survival plot for CCNG2 in prostate cancer suggests that low CCNG2 expression is linked with diminished survival rates in PCa over time (Fig. 4H) suggesting that CCNG2 is part of a tumor suppressive program induced by SAL.

Since the KD of BHLHE40 significantly reduces the *CCNG2* mRNA (Fig. 4I) it suggests that BHLHE40 up-regulates CCNG2 expression and the atypical CCNG2 is part of the BHLHE40 tumor suppressive pathway. Vice versa, the CCNG2 KD leads to a significant reduction in the expression of *BHLHE40* mRNA and protein (Fig. 4J, K). These data suggest a reciprocal control loop between BHLHE40 and CCNG2. Accordingly, the decrease in cellular senescence by CCNG2 KD is associated with decline in *CDKN2B* (Fig. 4L). The induction of p21^WAF1/Cip1^, by SAL was reversed after CCNG2 KD (Fig. 4K) supporting that CCNG2 regulates cellular senescence. In accordance with the results of BHLHE40 KD in the C4-2 cell line, the phosphorylation of AKT at S473 was strongly induced after CCNG2 KD but no changes were detected in the phospho-p70S6K (Fig. 4M). This indicates that CCNG2 represses p-AKT level and further confirms that the regulation of cellular senescence is not only mediated by p-AKT levels but rather through an alternative pathway. Collectively, these data indicate that CCNG2 and BHLHE40 act in tandem to promote cellular senescence in the AR signaling.

### BHLHE40 overexpression enhances cellular senescence in CSPC and CRPC

**Fig. 5:**
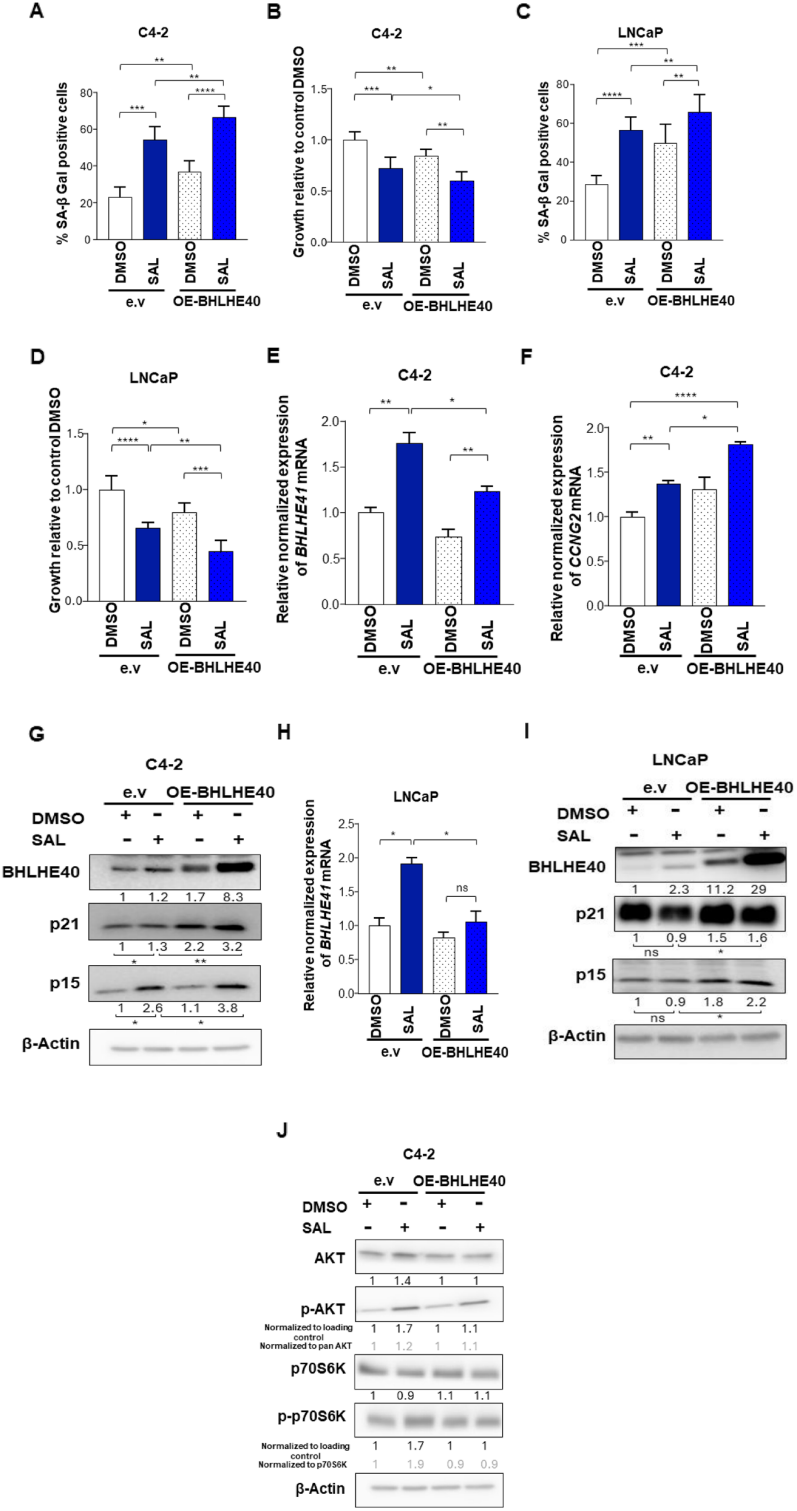
BHLHE40 overexpression induces cellular senescence. **A** and **C** Senescence assay shows further induction in cellular senescence by overexpression of BHLHE40 in both C4-2 and LNCaP cell lines (*n* = 3). **B** and **D** Crystal violet staining to analyze the growth of BHLHE40 overexpressed cells in both cell lines (*n* = 3). **E** *BHLHE41* mRNA level measured by qRT-PCR in C4-2 cell line (*n* = 3). **F** *CCNG2* mRNA level measured by qRT-PCR in C4-2 cell line (*n* = 3). **G** Western blot of treated C4-2 cells shows induction in p15 ^INK4b^ and p21 ^WAF1/Cip1^ after BHLHE40 overexpression (*n* = 3).** H** *BHLHE41* mRNA level measured by qRT-PCR in LNCaP cell line (*n* = 3).** I** Western blot of treated LNCaP cells reveals induction in p15 ^INK4b^ and p21 ^WAF1/Cip1^ after BHLHE40 overexpression (*n* = 3). **J** phosphorylation of AKT and p70S6K are backed to basal level after overexpression of BHLHE40. The ratio of p-AKT versus AKT and p70S6K versus p-p70S6K are indicated in grey. *P* value < 0.0001 = ****, 0.001 = ***, < 0.01 = **, < 0.05 = *

The functionality of *BHLHE40* overexpression plasmid (OE-BHLHE40) was shown by the repression of BHLHE*41* mRNA (Fig. 5E, H), induction of *CCNG2* expression, which is consistent with the reduction of *CCNG2* by BHLHE40 KD (Fig. 5F) and induction of senescent cell level (Fig. 5A, C, and S9A).

Surprisingly, for both KD and overexpression scenarios, a reduction in growth was observed (Fig. 5B, D, and S9B). These data suggest that not only does BHLHE40 act in the regulation of cellular senescence, but it might also play a role in other mechanisms in PCa cells including dormancy. Protein levels of p15^INK4b^ and p21^WAF1/Cip1^ were increased in BHLHE40-overexpressed cells (Fig. 5G, I). These data confirm the SA b-Gal activity assay. Concerning BHLHE40-overexpression analysis of phosphorylation of AKT and p70S6K suggests that induction of p-levels of both kinases by SAL are reduced (Fig. 5J, S10). Thus, these data confirm the induction of AKT and p70S6K phosphorylation by BHLHE40 KD suggesting that the BHLHE40 mediated cellular senescence especially under SAL treatment is associated with a pathway that leads to suppression of AKT and p70S6K phosphorylation and thus provides a novel AKT-alternative pathway to control cellular senescence in PCa.

### Co-immunoprecipitation (Co-IP) assays revealed interaction between AR and BHLHE40 in both CSPC and CRPC cell lines

**Fig. 6:**
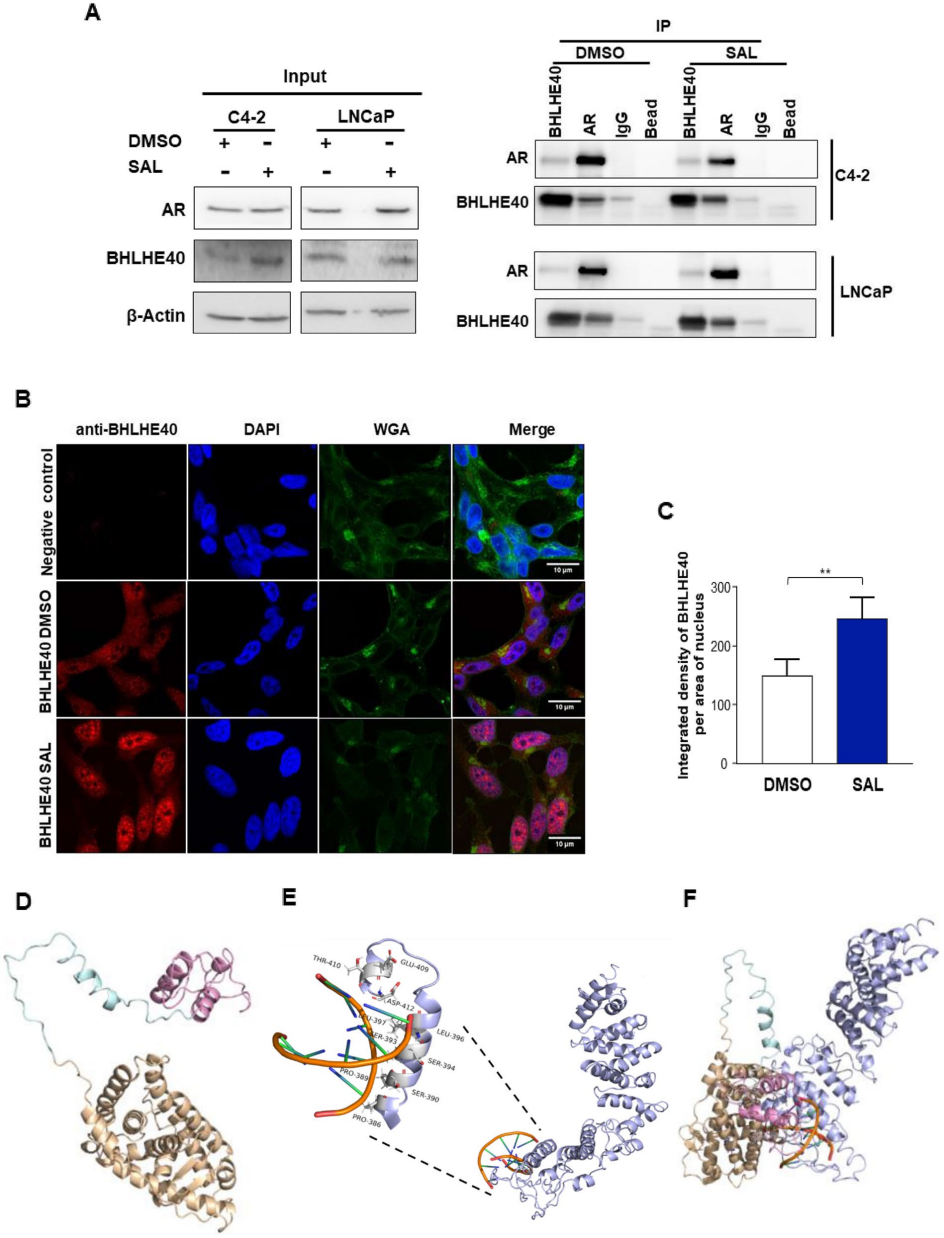
BHLHE40 interacts with AR and SAL induces BHLHE40 translocation to the nucleus. **A** Co-Immunoprecipitation assay for both AR and BHLHE40 confirms interaction between AR and BHLHE40 in both cell lines (*n* = 2). **B** Immunofluorescent staining indicates an increase in the translocation of BHLHE40 to the nucleus after SAL treatment in C4-2 cell line (*n* = 2). DAPI was used to stain nucleus and wheat germ agglutinin (WGA) was used to detect cell membrane. **C**Quantification of the integrated density of BHLHE40 in the nucleus. **D** AR DBD-hinge-LBD region prediction model. The AR–DBD domain from amino acids (556 – 623) showed in pink, hinge region from amino acids (624 – 665) depicted in pale cyan and AR-LBD domain consider from amino acids (666 – 919) is with wheat color. C-Score = -0.20, TM- Score = 0.69 -/ + 0.12 (**E**) BHLHE40 prediction model is shown in light blue color. Interaction of BHLHE40 with DNA was evaluated by docking. BHLHE40 binds DNA from the area containing amino acids (384–412). C-Score = -1.10, TM- Score = 0.58 -/ + 0.14 and docking score: 8354, ACE: **-**540.31. **F** BHLHE40-AR interaction prediction was performed by docking. Docking score: 15,216, ACE: -539.63

To investigate the potential interaction between endogenous BHLHE40 and the endogenous AR, Co-IP experiments were conducted with endogenous factors on both cell lines. BHLHE40 was detected in the immunoprecipitated AR and vice versa, under both DMSO or SAL treatments, indicating a protein–protein complex of AR with BHLHE40 (Fig. 6A). Additionally, immunofluorescence staining was performed to view the intracellular localization of BHLHE40 with and without androgen treatment (Fig. 6B, C and S11A, B). Interestingly, the data suggest that SAL promotes the translocation of BHLHE40 into the nucleus suggesting a co-translocation of AR and BHLHE40 by SAL. As BHLHE40 is a transcription factor, this translocation potentially regulates downstream targets through genomic regulation.

Based on this, we hypothesized that the reads obtained by AR-ChIP-seq also contain bHLH motifs. Interestingly, motif analysis for AR ChIP-seq data of C4-2 cells treated with SAL revealed many AR binding sites that also contain a bHLH motif in reads. Focusing on genes, it revealed around 2400 genes with both AR and bHLH binding sites, which may explain the observed large overlap of DEGs between these two transcription factors supporting our hypothesis. With pathway analysis of this gene set, the pathways of Circadian rhythm, Heme signaling, BMAL1-Clock activates circadian gene expression, and cellular senescence emerged (Table S5). This suggests a link between androgen signaling and circadian rhythm and cellular senescence.

To assess potential interactions between AR and BHLHE40, protein structure predictions and protein–protein docking modeling were conducted for the AR-DBD-hinge-LBD region with BHLHE40 protein. The 3D AR-DBD-hinge-LBD region prediction is depicted in (Fig. 6D, S12A), while the BHLHE40 structure and the region interacting with DNA, along with the involved amino acids, are illustrated in (Fig. 6E). Both AR and BHLHE40 structures exhibit high confidence scores and topology with a strong score for AR DBD-hinge-LBD and BHLHE40. The docking predictions show a high score for the interaction between AR and BHLHE40-bound DNA (Fig. 6F, S12B). The modeling suggests AR interacts with BHLHE40 through its ligand binding domain and that DNA binding domains of both proteins are positioned near each other.

## Discussion

Interestingly, the AR has oncogenic and tumor suppressive activity [60]. It has been observed that supraphysiological androgen levels exhibit inhibitory effects on tumor growth, especially in PCa cells that thrive in low androgen environments [5]. In addition, our SAL treated C4-2 xenografted mice results show the inhibitory effect of SAL on tumor growth. These data suggest that SAL triggers tumor suppressive program of the AR. Of note, the AR is also a tumor suppressive in AR and estrogen receptor positive breast cancer cells [61]. Mechanistically, in PCa the AR regulates target genes that control cell cycle such as SPOP or LRIG [62, 63] or interacts with tumor suppressors [40]. Also, the tumor suppressor pRb mediates growth inhibition by SAL and sensitizes CRPC to SAL treatment [33] being in line with previous observations by Gao et al. [64].

As a cellular response it was shown that SAL induces cellular senescence in an AR dependent manner in both CSPC and CRPC. Senescence induction by SAL was confirmed in ex vivo treated human prostatectomy samples [3, 11] and in our C4-2 xenograft mice experiments. Cellular senescence is characterized by a stable arrest of cells. It is known that senescence cells exhibit the senescence-associated secretory phenotype with secretion of cytokines and chemokines. Interestingly, recently it was shown that BAT induces pro-inflammatory changes of gene expression and that BAT treatment augments antitumor immune responses associated with beneficial clinical response of patients [65].

The detailed molecular pathway of AR mediated cellular senescence remains open. Our data suggest that BHLHE40 mediates SAL-induced cellular senescence. BHLHE40 is a member of the circadian gene family and is responsible for regulating circadian rhythms [66, 67] and functions as a transcription regulator [68]. However, the role of BHLHE40 in PCa remained underexplored.

Our data further indicate that *BHLHE40* gene is a direct target of AR, which is upregulated by SAL and that BHLHE40 protein interacts with AR directly or indirectly in both CSPC and CRPC cell lines. Protein structure predictions suggest rather a direct AR-BHLHE40 interaction with a high score between the LBD of AR and BHLHE40 supported by the co-translocation of BHLHE40 to the nucleus by SAL. This finding may explain the large overlap of genes with the SAL transcriptome landscape. The KD of BHLHE40 has no detectable effect on AR protein level as well as no significant changes in the mRNA level of SAL-induced direct AR target genes indicating that the AR at SAL rather regulates BHLHE40 target genes. In line with this, GSEA indicates that the KD of BHLHE40 only slightly changes the hallmark of androgen response genes (data not shown). These observations suggest that the impact of BHLHE40 to co-regulate AR target genes is minor but may be specific for a particular AR signaling pathway. The transcriptome data analyses also suggest that only a subset of AR function, that is the SAL-induced cellular senescence, is mediated by BHLHE40. Thus, it seems that BHLHE40 mediates only a specific AR controlled pathway to induce SAL-mediated cellular senescence.

In esophageal cancer it was shown that BHLHE40 can induce cellular senescence [68]. Of note, thyroid hormone-induced cellular senescence is mediated in part by BHLHE40 [16]. However, the mechanisms through which BHLHE40 mediates cellular senescence remains unclear. Our data propound that SAL-induces BHLHE40 activity to mediate SAL-induced cellular senescence in 2D and 3D cell cultures. Previous studies have shown that SAL activates the AKT pathway leading to increased phosphorylation of AKT that mediates in part cellular senescence [5, 11]. However, BHLHE40 KD and overexpression experiments suggest that BHLHE40 is a mediator of cellular senescence with rather inhibiting the phosphorylation of AKT. This implies that BHLHE40 mediates SAL-induced cellular senescence through a novel, alternative AKT pathway.

One potential pathway identified here is that CCNG2 as part of the FOXO-mediated transcription pathway, which is linked to one of the AR pathways in PCa [69, 70]. CCNG2, is an unconventional cyclin which partly through p21^WAF1/Cip1^ negatively regulates the cell cycle [71], was found to be a gene in the FOXO-mediated transcription pathway in our BHLHE40 KD RNA-seq. It is known that CCNG2 represses AKT phosphorylation and activation, and vice versa [22, 72]. Our data show that KD of CCNG2 using siRNA induces phosphorylation of AKT, like the effect of BHLHE40 KD, supporting the evidence of a novel AKT alternative pathway to mediate SAL-induced cellular senescence. Moreover, there appears to be a reciprocal regulation between CCNG2 and BHLHE40, as CCNG2 KD reduces the level of BHLHE40.

BHLHE40 is known as a circadian rhythm factor and circadian repressor [73]. Interestingly, many genes regulating circadian rhythm are aberrantly expressed in cancer [74]. Although the role of circadian rhythm in PCa risk is unclear, there is some evidence that individuals who suffer from sleep disorders or worknight shift are at a higher risk of developing PCa, which is suggested to be linked to c-Myc expression [21]. Recently, it was shown that epigenetic activity reprograms circadian rhythm associated with androgen-independent PCa [75]. We show here that BHLHE40 is downregulated in PCa tumor samples and low expression is linked to lower survival of PCa patients. This suggests that either BHLHE40 has independently of regulating circadian rhythm a tumor suppressive function or alternatively, that a proper circadian rhythm is part of a tumor suppressive program in cancer. Our data demonstrate that AR and BHLHE40 interact in a protein complex and co-translocate to the nucleus. In line with this, bioinformatic analyses of AR ChIP-seq suggests that many AR binding sites in chromatin contain bHLH binding motifs. Notably, genes containing those motifs are in pathways regulating circadian rhythm. Thus, this study offers a link between androgen signaling and circadian rhythm.

One tumor suppressive pathway by BHLHE40 identified here is the regulation of cellular senescence and evidence of other types of dormancy [51]. The findings from our BHLHE40 study suggest that BHLHE40 mediates SAL-induced cellular senescence in PCa cell lines through the regulation of CCNG2. In fact, SAL leads to upregulation of BHLHE40 mRNA and protein. BHLHE40 interacts with AR and mediates cellular senescence. Cyclin G2 is a positive downstream target of BHLHE40 to mediate cellular senescence proposing a novel AR-BHLHE40-CyclinG2 axis.

## Conclusions

**Fig. 7:**
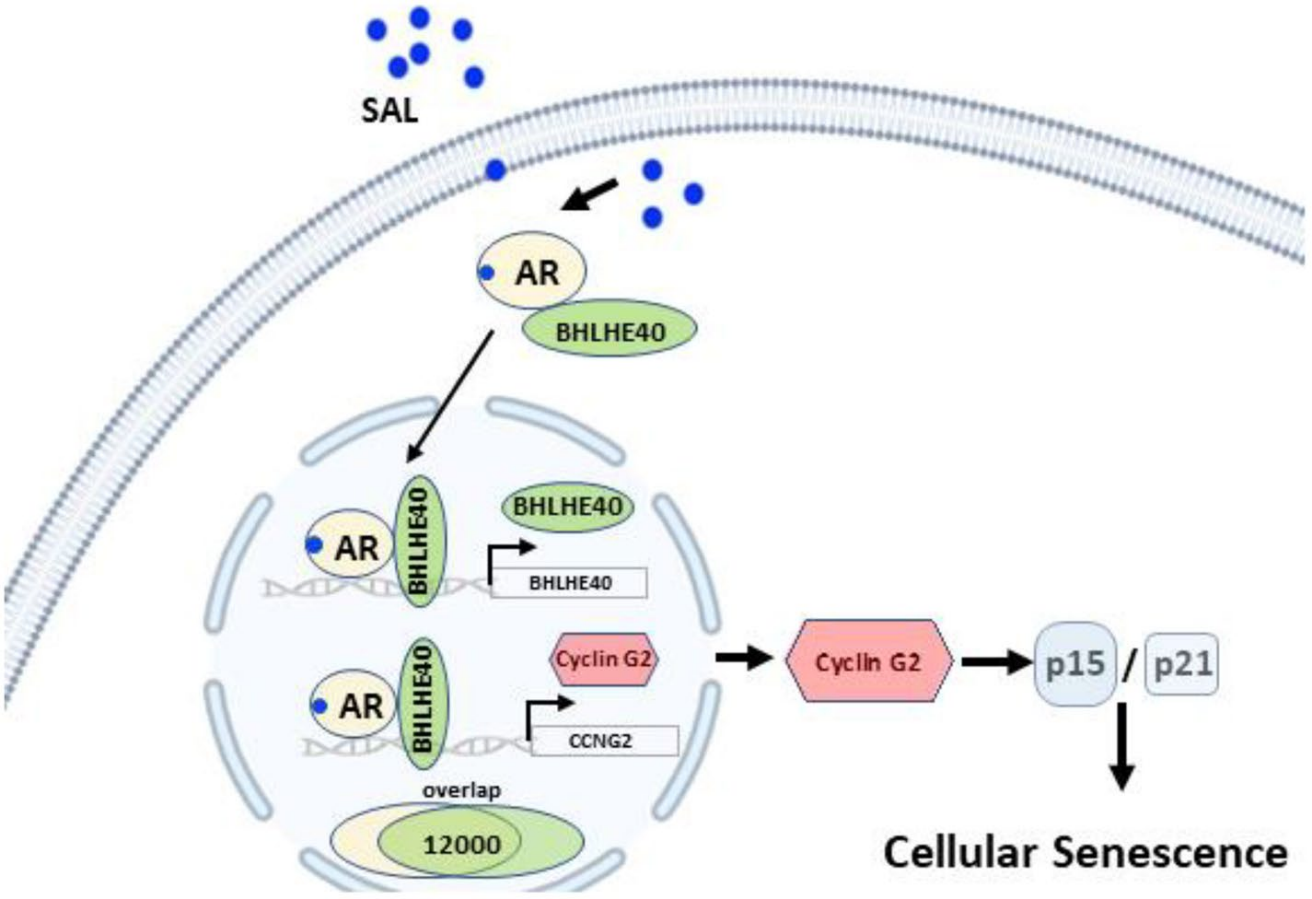
BHLHE40 mediates cellular senescence via novel tumor suppressive axis androgen receptor-BHLHE40-Cyclin G2. The figure was generated in *Biorender* and modified

Androgen treatment used in bipolar androgen therapy induces both the expression and translocation of BHLHE40 into the nucleus. BHLHE40 encoding DEC1 mediates cellular senescence by its direct target gene CCNG2, that encodes the atypical and tumor suppressive Cyclin G2, as a novel tumor suppressive axis encompassing androgen receptor-BHLHE40-Cyclin G2 in order to mediate cellular senescence in PCa cells (Fig. 7).

### Supplementary Information

Supplementary Material 1.

Supplementary Material 2.

## Data Availability

The AR ChIP-Seq dataset analyzed during the current study is available in the Gene Expression Omnibus (GEO) with accession number GSE179684. The BHLHE40 ChIP-Seq dataset analyzed during the current study is available in the GEO with accession number GSE137848. The prostatectomy samples RNA-Seq datasets analyzed during the current study are available in the GEO accession number GSE89223 and GSE179321.The RNA-seq data generated in this study are available in GEO with accession number GSE262117. The Venn diagram’s gene lists from this study are listed in supplementary excel file. All R script codes used during this study are available in the supplementary data.

## Abbreviations

AR: Androgen receptor
BAT: Bipolar androgen therapy
BHLHE40: Basic helix loop helix e40
CCNG2: Cyclin G2
CSPC: Castration sensitive prostate cancer
CRPC: Castration resistant prostate cancer
DEG: Differentially expressed genes
GSEA: Gene set enrichment analysis
SAL: Supraphysiological androgen level
Co-IP: Co-immunoprecipitation
KD: Knockdown
OE: Overexpression
s.c: Subcutaneously
SA β-Gal: Senescence-associated beta galactosidase
