# Supplementary material for: The clock gene BHLHE40 and atypical CCNG2 control androgen-induced cellular senescence as a novel tumor suppressive pathway in prostate cancer
Source: J Exp Clin Cancer Res. 2024 Jun 20;43:174. doi: 10.1186/s13046-024-03097-6 (PMC11188219; doi:10.1186/s13046-024-03097-6)
Supplement: Supplementary file 1 — Supplementary Material 1. [file 13046_2024_3097_MOESM1_ESM.pdf]

A

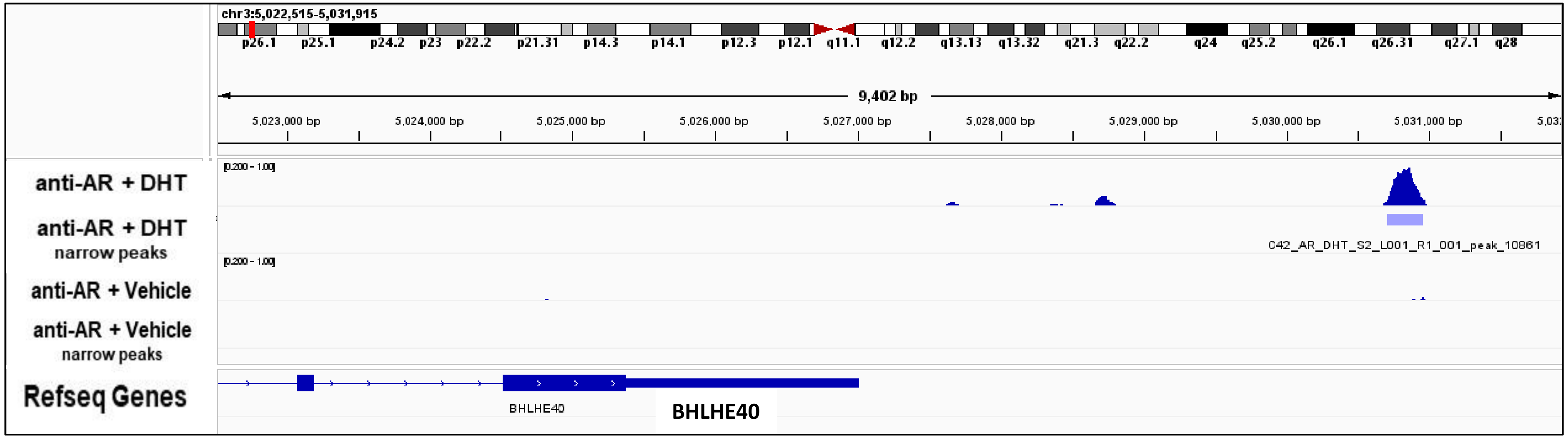

chr3:5,022,515-5,031,915
p26.1 p25.1 p24.2 p23 p22.2 p21.31 p14.3 p14.1 p12.3 p12.1 q11.1 q12.2 q13.13 q13.32 q21.3 q22.2 q24 q25.2 q26.1 q26.31 q27.1 q28
9,402 bp
5,023,000 bp 5,024,000 bp 5,025,000 bp 5,026,000 bp 5,027,000 bp 5,028,000 bp 5,029,000 bp 5,030,000 bp 5,031,000 bp
anti-AR + DHT
anti-AR + DHT narrow peaks
C42_AR_DHT_S2_L001_R1_001_peak_10861
anti-AR + Vehicle
anti-AR + Vehicle narrow peaks
Refseq Genes
BHLHE40
BHLHE40

B

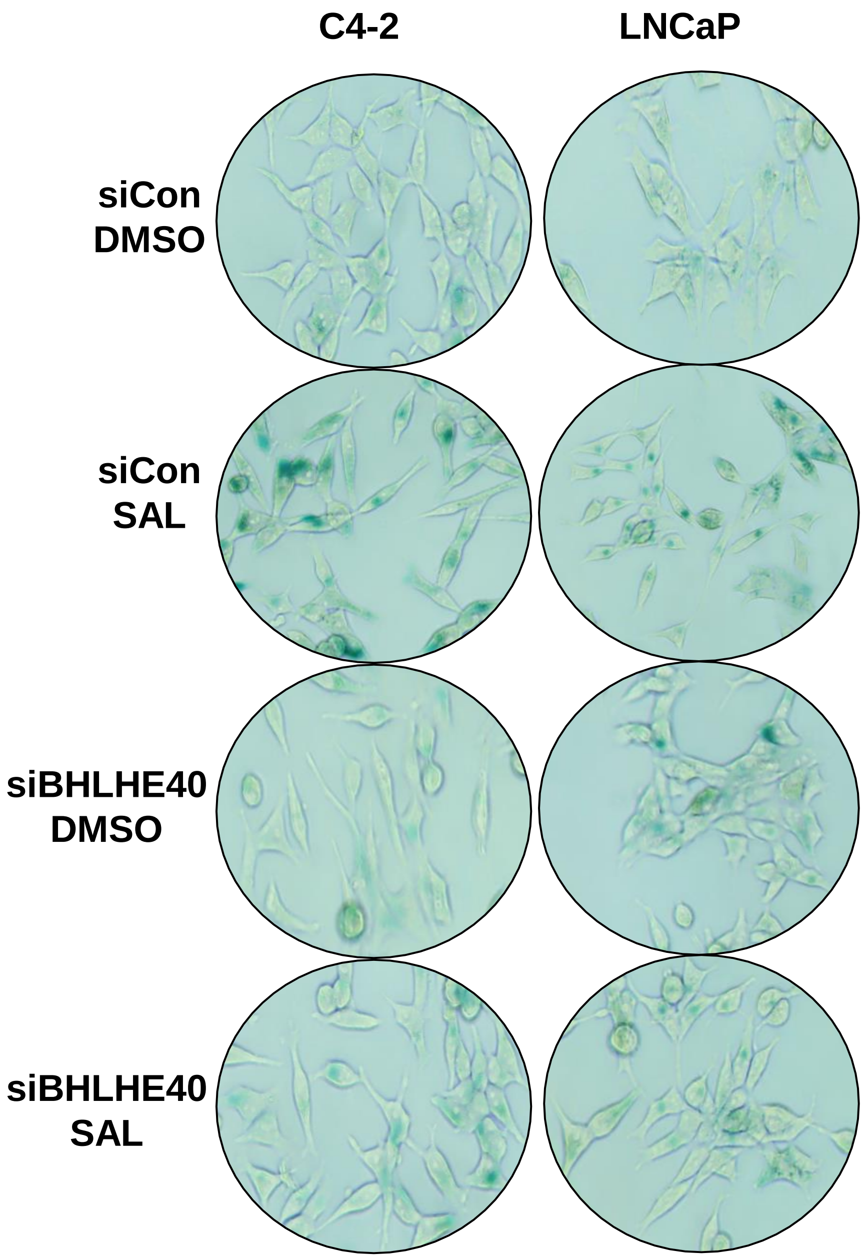
C4-2
LNCaP
siCon
DMSO
siCon
SAL
siBHLHE40
DMSO
siBHLHE40
SAL

C

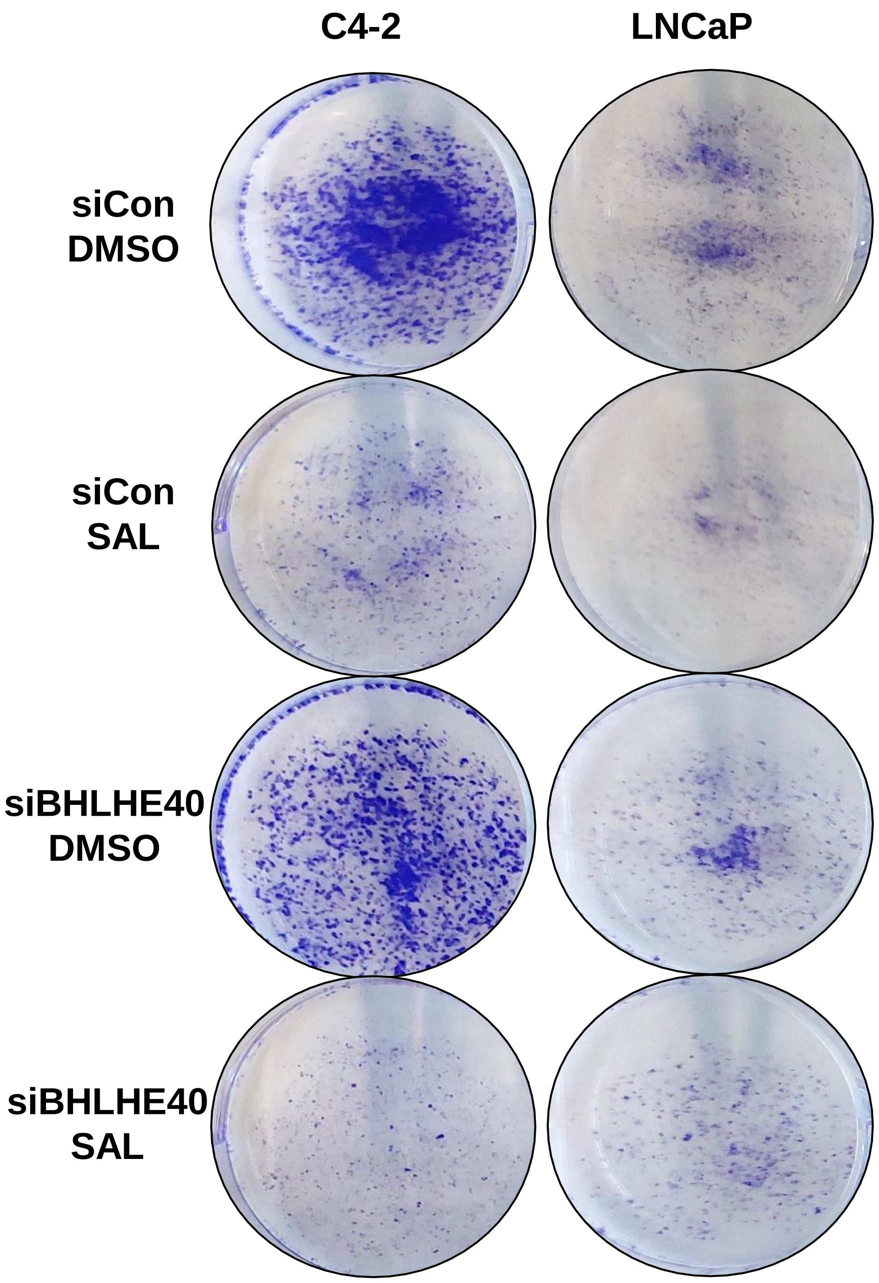
C4-2
LNCaP
siCon
DMSO
siCon
SAL
siBHLHE40
DMSO
siBHLHE40
SAL

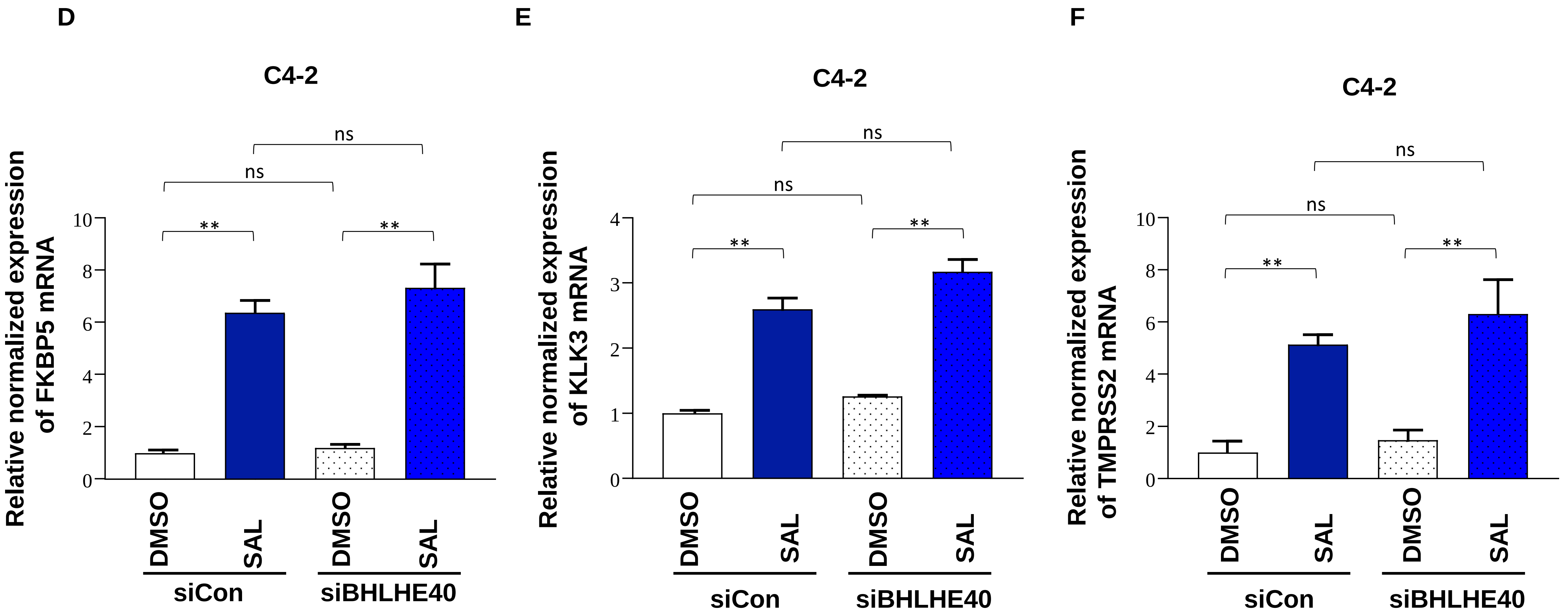
D
C4-2
Relative normalized expression of FKBP5 mRNA
ns
ns
**
**
10
8
6
4
2
0
DMSO
SAL
DMSO
SAL
siCon
siBHLHE40
E
C4-2
Relative normalized expression of KLK3 mRNA
ns
ns
**
**
4
3
2
1
0
DMSO
SAL
DMSO
SAL
siCon
siBHLHE40
F
C4-2
Relative normalized expression of TMPRSS2 mRNA
ns
ns
**
**
10
8
6
4
2
0
DMSO
SAL
DMSO
SAL
siCon
siBHLHE40

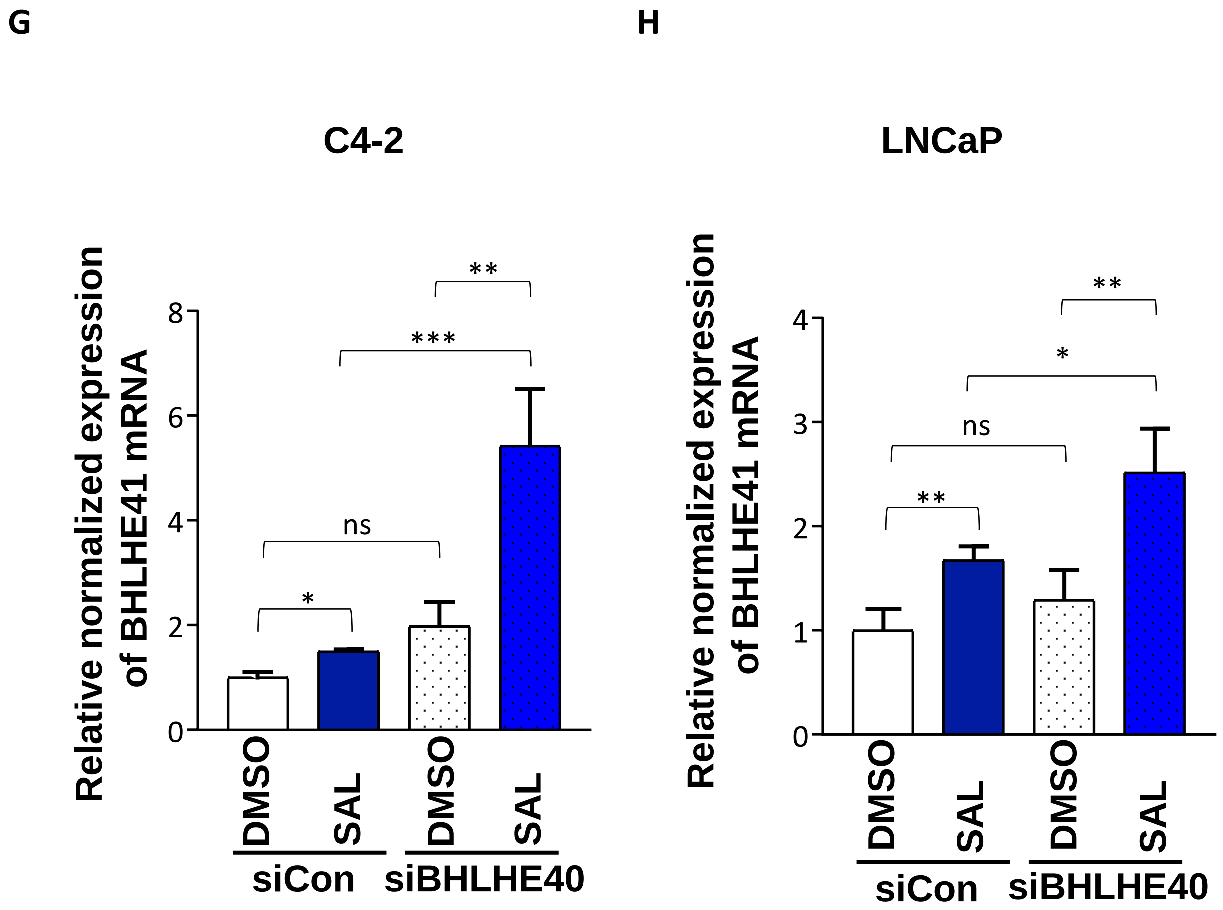

Fig. S1- AR regulates BHLHE40 expression whereas the expression of AR target genes is not significantly affected by the BHLHE40 KD. A: ChIP-seq of SAL treated C4-2 cells revealed an AR binding site downstream of *BHLHE40* gene. Several ARE binding sites are unveiled in the analyzed peak sequence. IGV software was used to visualize the peak region. **B:** Pictures of SA β-Gal activity of treated cells for 72 h. **C:** Representative crystal violet staining**.** qRT-PCR of BHLHE40 KD cells treated with SAL or DMSO does not show changes in the androgen-induced expression of AR target genes **D:** *FKBP5* mRNA, **E:** *KLK3* mRNA and **F:** *TMPRSS2* mRNA. **G** and **H:** *BHLHE41* mRNA levels by BHLHE40 KD in C4-2 and LNCaP cells respectively. P value <0.01 = **, ns= non-significant.

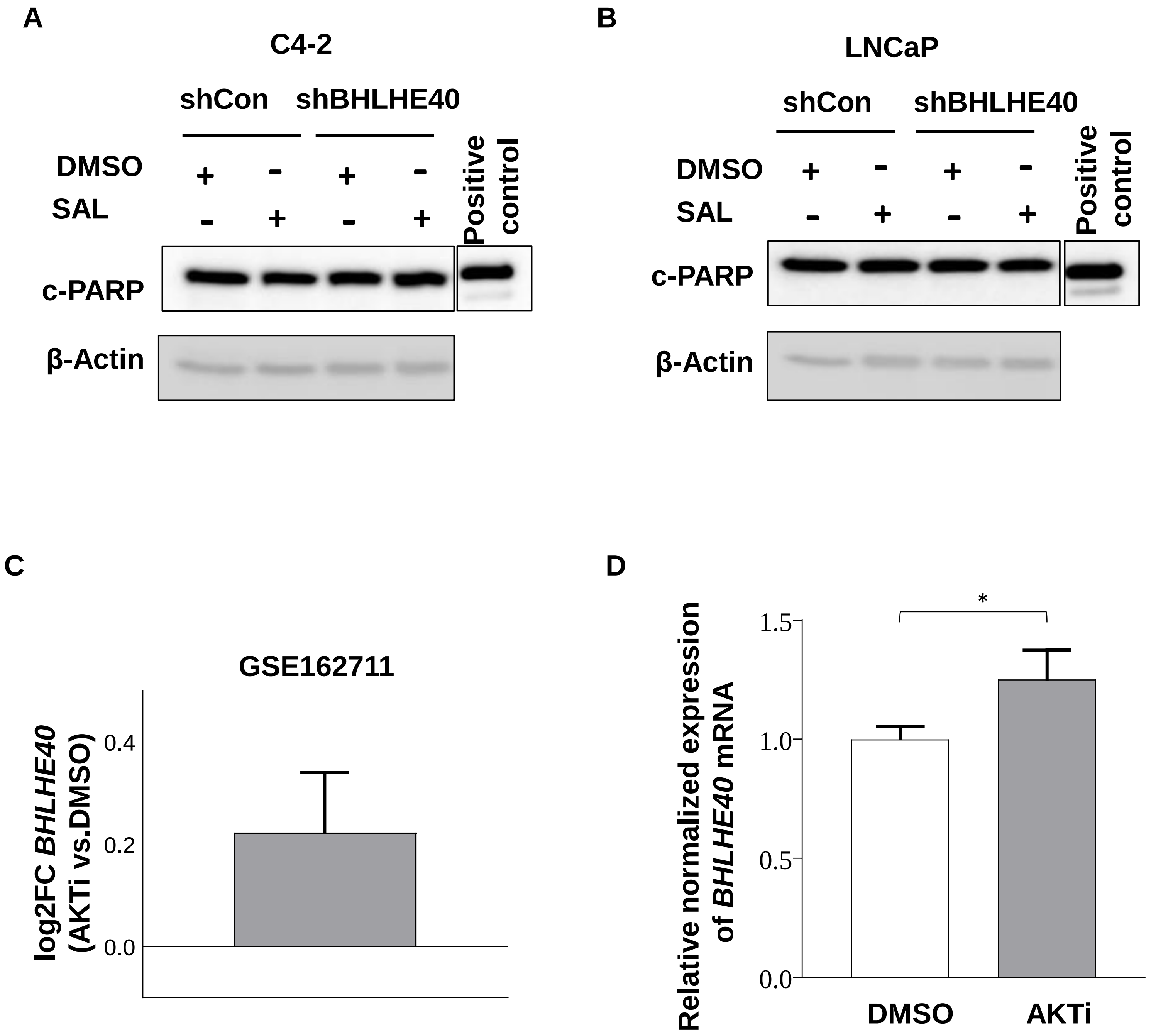

Fig. S2- BHLHE40 KD does not induce apoptosis and AKTi induces *BHLHE40* level. A and **B:** Western blot of C4-2 and LNCaP cells for c-PARP. Knock down of BHLHE40 does not show cleaved PARP as a marker of cell apoptosis. **C:** RNA-Seq of C4-2 cells treated with AKTi indicates slight up regulation of *BHLHE40*. **D:** qRT-PCR of C4-2 cells treated with AKTi showed induction of *BHLHE40* mRNA (n= 3). P value <0.05 = *.

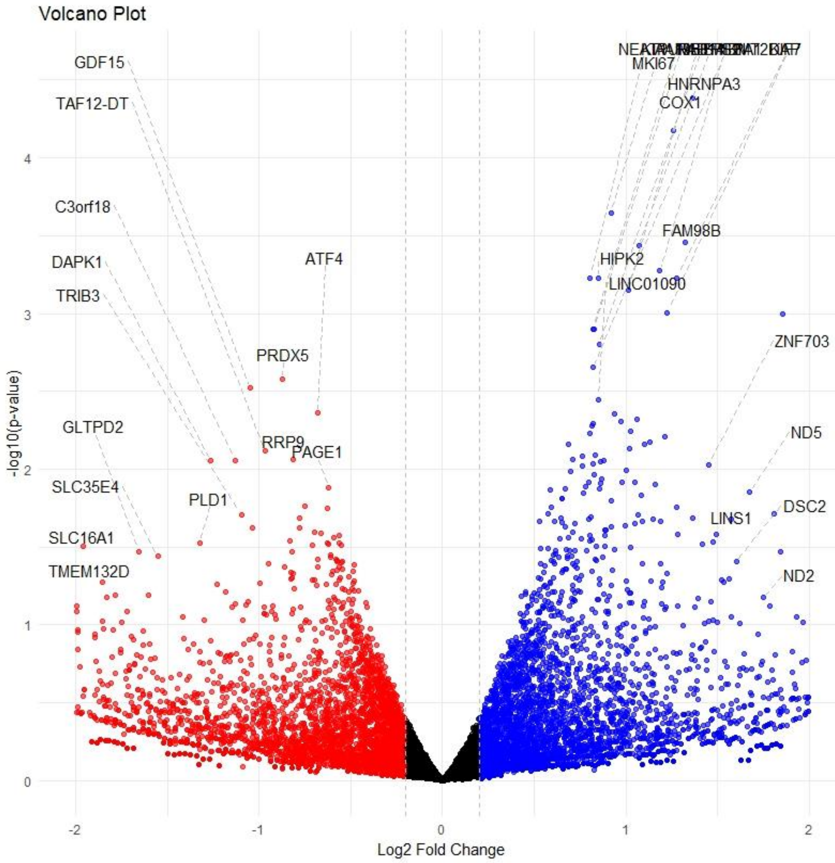

Fig. S3- Volcano plot for DMSO-treated BHLHE40 KD samples vs. relevant control. Volcano plot depicts overall expression changes by BHLHE40 KD samples treated with DMSO versus control samples with DMSO treatment (log2FC value was used for plotting).

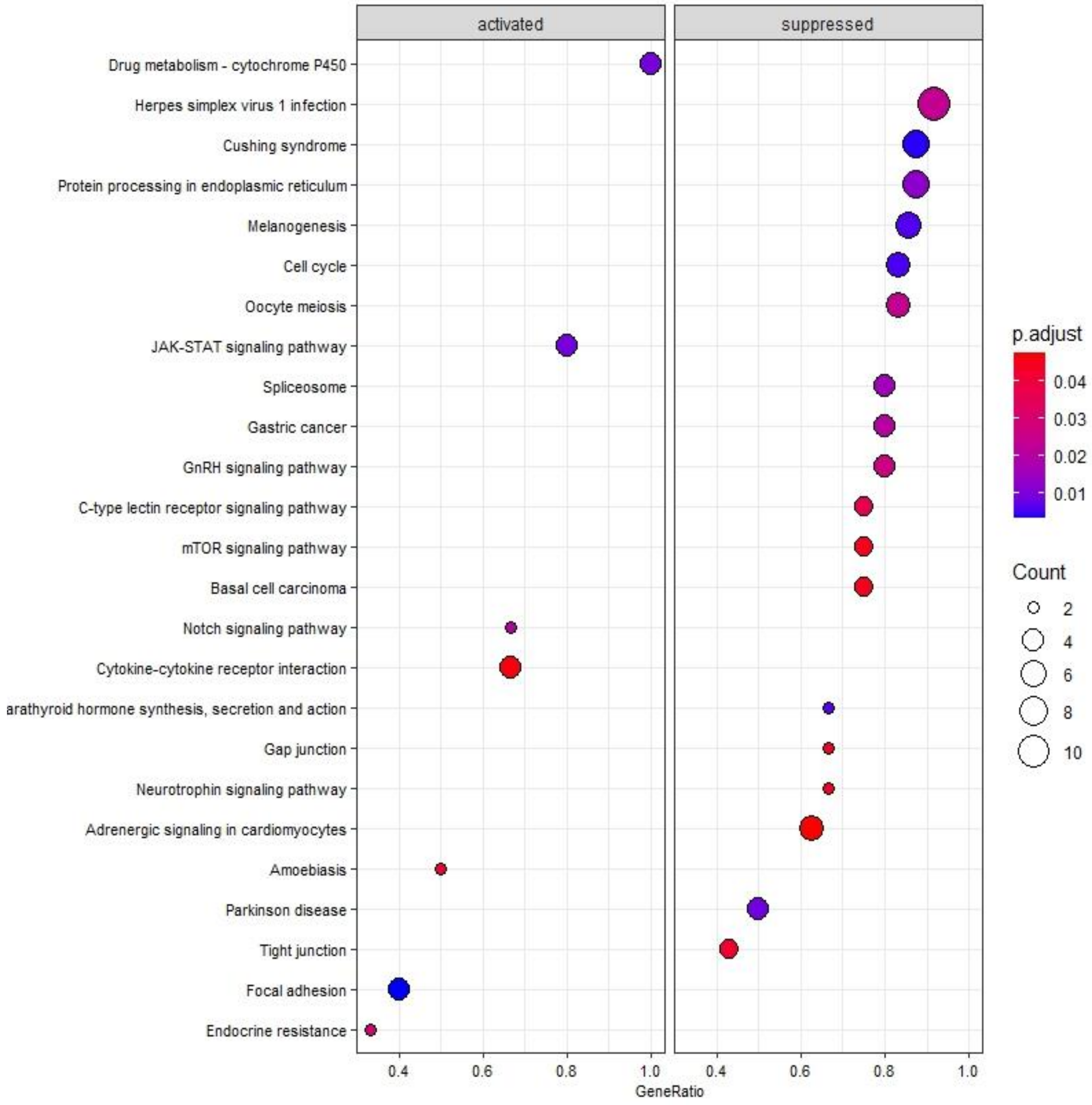

Fig. S4- Pathway analysis for specific gene from BHLHE40 KD SAL-treated. 810 genes from Fig. 3I and 147 genes from Fig. 3J were used for performing pathway analysis. Regulation of pathways are shown. Count refer to the number of genes in each pathway.

Fig. S5

A

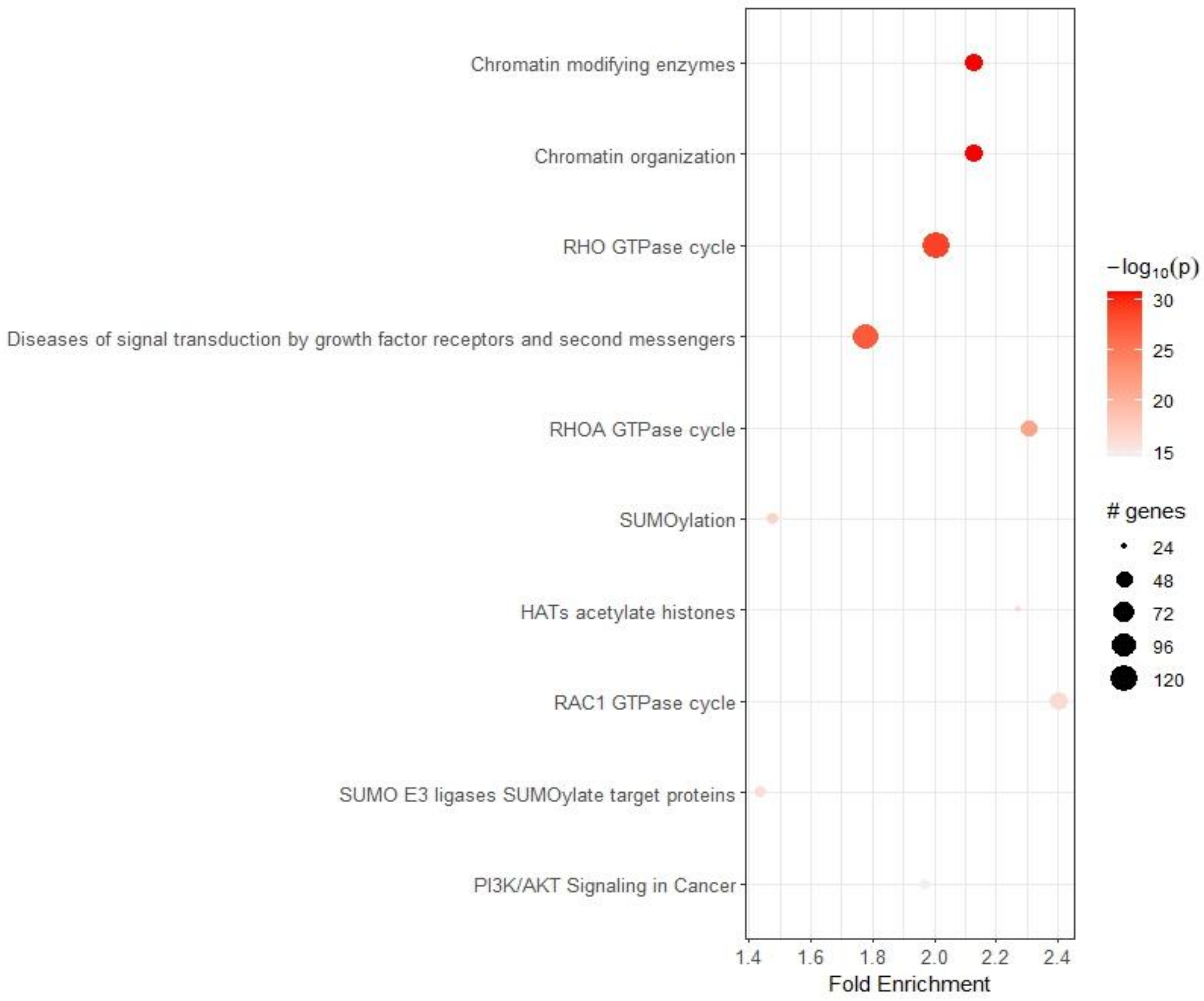
Chromatin modifying enzymes
Chromatin organization
RHO GTPase cycle
Diseases of signal transduction by growth factor receptors and second messengers
RHOA GTPase cycle
SUMOylation
HATs acetylate histones
RAC1 GTPase cycle
SUMO E3 ligases SUMOylate target proteins
PI3K/AKT Signaling in Cancer
1.4
1.6
1.8
2.0
2.2
2.4
Fold Enrichment
$-\log_{10}(p)$
30
25
20
15
genes
24
48
72
96
120

Fig. S5

B

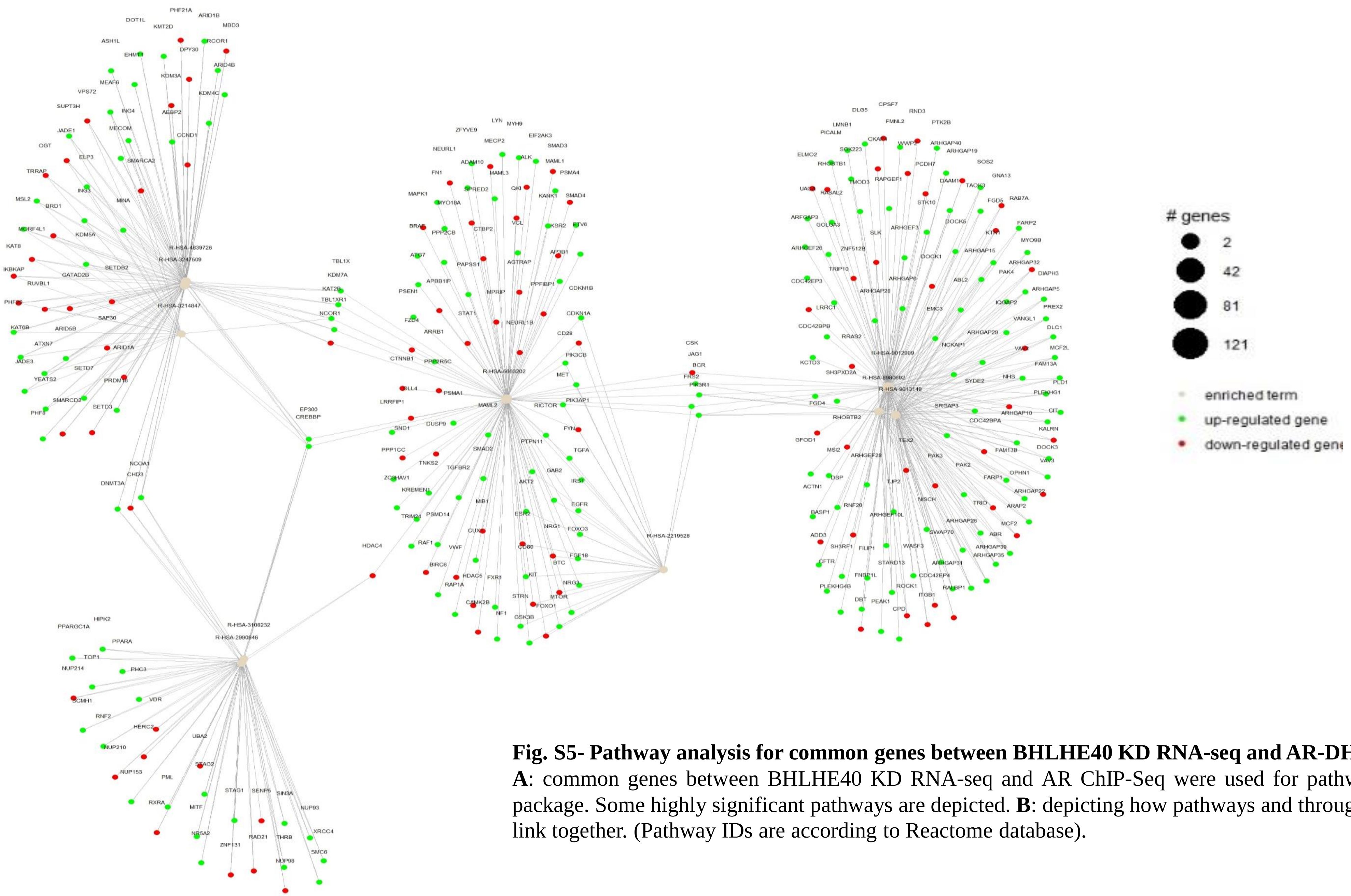

Fig. S5- Pathway analysis for common genes between BHLHE40 KD RNA-seq and AR-DHT ChIP-seq.
A: common genes between BHLHE40 KD RNA-seq and AR ChIP-Seq were used for pathway analysis by R package. Some highly significant pathways are depicted. **B**: depicting how pathways and through which genes are link together. (Pathway IDs are according to Reactome database).

A

Matrix ID	Name	Score	Relative score	Sequence ID	Start	End	Strand	Predicted sequence
MA0464.3	MA0464.3.BHLHE40	13.552525	0.9971479240834746	CCNG2promoter	1970	1977	+	tcacgtgc
MA0464.3	MA0464.3.BHLHE40	13.400488	0.9944694511588582	CCNG2promoter	1970	1977	-	gcacgtga
MA0464.2	MA0464.2.BHLHE40	14.630148	0.9909467801334144	CCNG2promoter	1969	1978	+	gtcacgtgcc
MA0464.2	MA0464.2.BHLHE40	14.478108	0.9884209868071893	CCNG2promoter	1969	1978	-	ggcacgtgac
MA0464.2	MA0464.2.BHLHE40	4.4724283	0.8221992132243172	CCNG2promoter	770	779	+	accacatgcc
MA0464.3	MA0464.3.BHLHE40	3.4692159	0.8195074253091997	CCNG2promoter	1344	1351	-	ccacggga
MA0464.3	MA0464.3.BHLHE40	3.0136213	0.8114810875568892	CCNG2promoter	771	778	+	ccacatgc
MA0464.2	MA0464.2.BHLHE40	3.4207733	0.8047283399986008	CCNG2promoter	1343	1352	+	ctcccgtggc

B

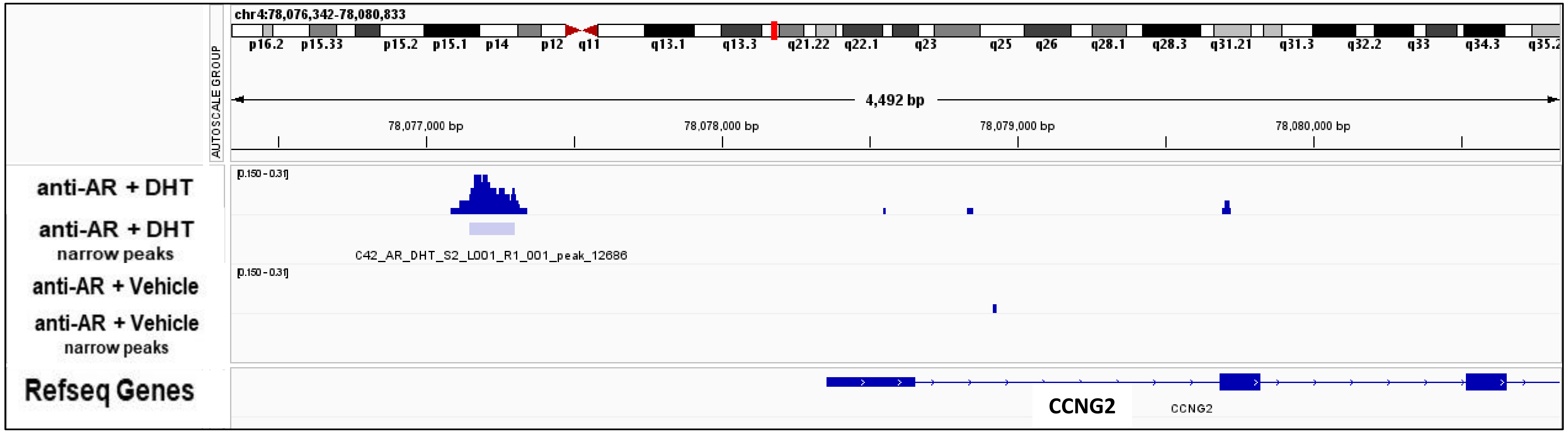

Fig. S6- ChIP-seq shows AR binding site upstream of *CCNG2*. A: Predicted binding sites for BHLHE40 in the promoter of CCNG2 shown in Fig. 4A. JASPAR-2022 was used for motif binding prediction. In the close promoter region of CCNG2, motif binding site of BHLHE40 transcription factor was found with high score. **B:** ChIP-seq of C4-2 cells treated with SAL revealed an AR binding site upstream of *CCNG2* gene. IGV software was used to visualize the peak region.

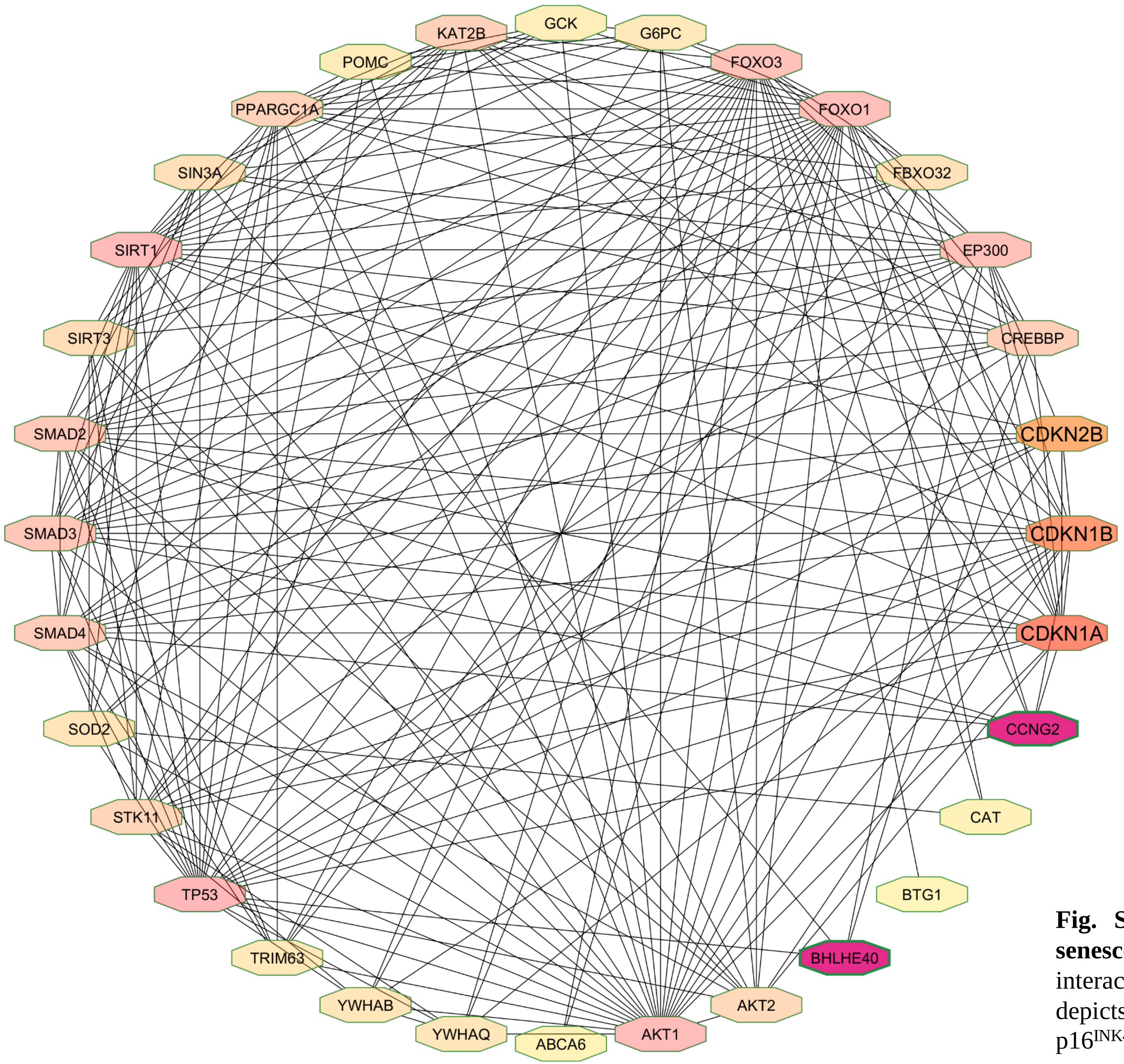

Fig. S7: Network interaction of BHLHE40 and CCNG2 with some cellular senescence markers. Cytoscape software was utilized to create the network showing the interaction between CCNG2 and BHLHE40 with cellular senescence markers. Graph depicts known network interactions between BHLHE40 and CCNG2 with $p15^{INK4b}$, $p16^{INK4a}$ and $p21^{WAF1/Cip1}$.

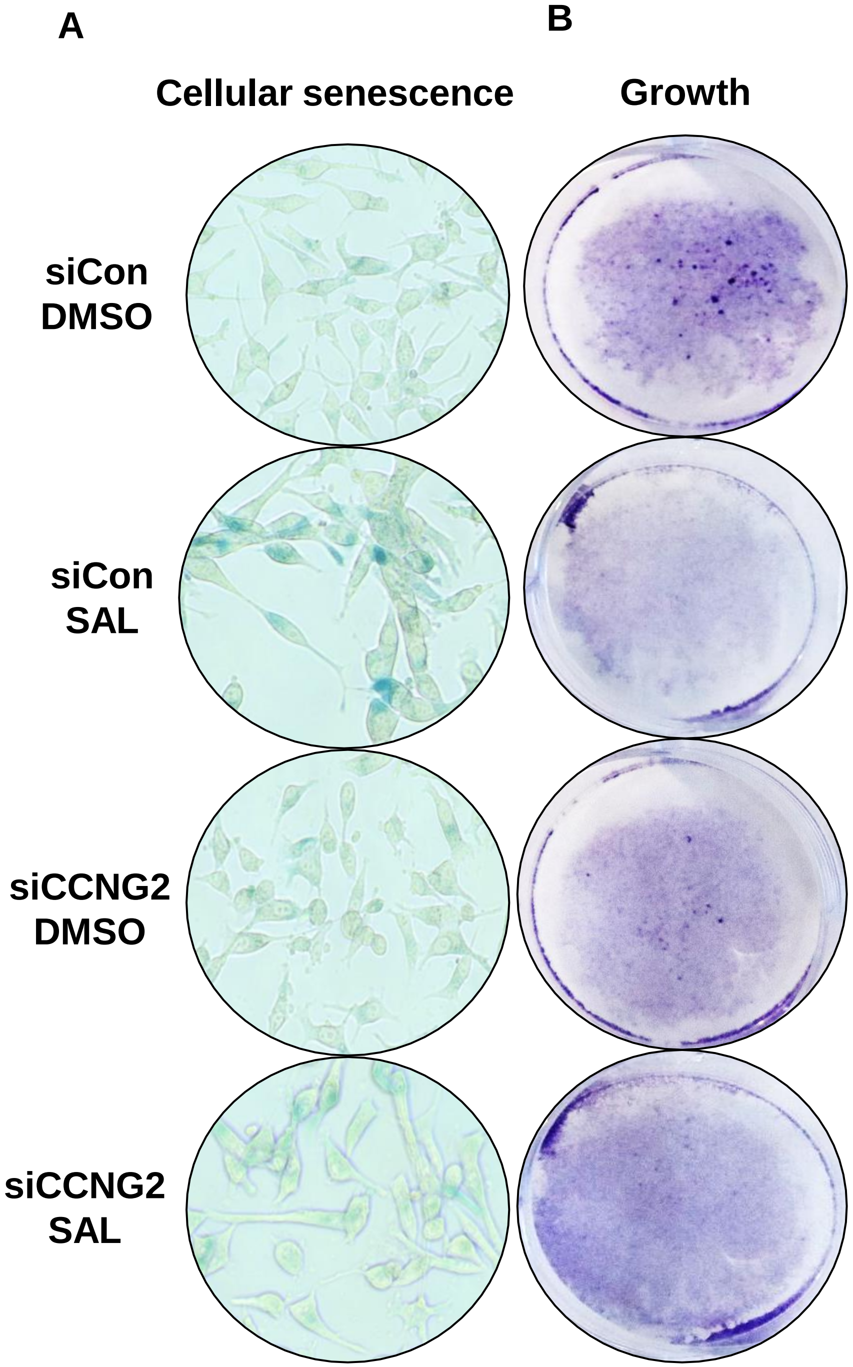

Fig. S8: CCNG2 KD reduces cellular senescence in C4-2 cell line. A: Detection of SA β-Gal activity of treated cells for 72 h. **B:** Representative crystal violet staining showed induction of growth by CCNG2 KD at SAL in C4-2 cell line.

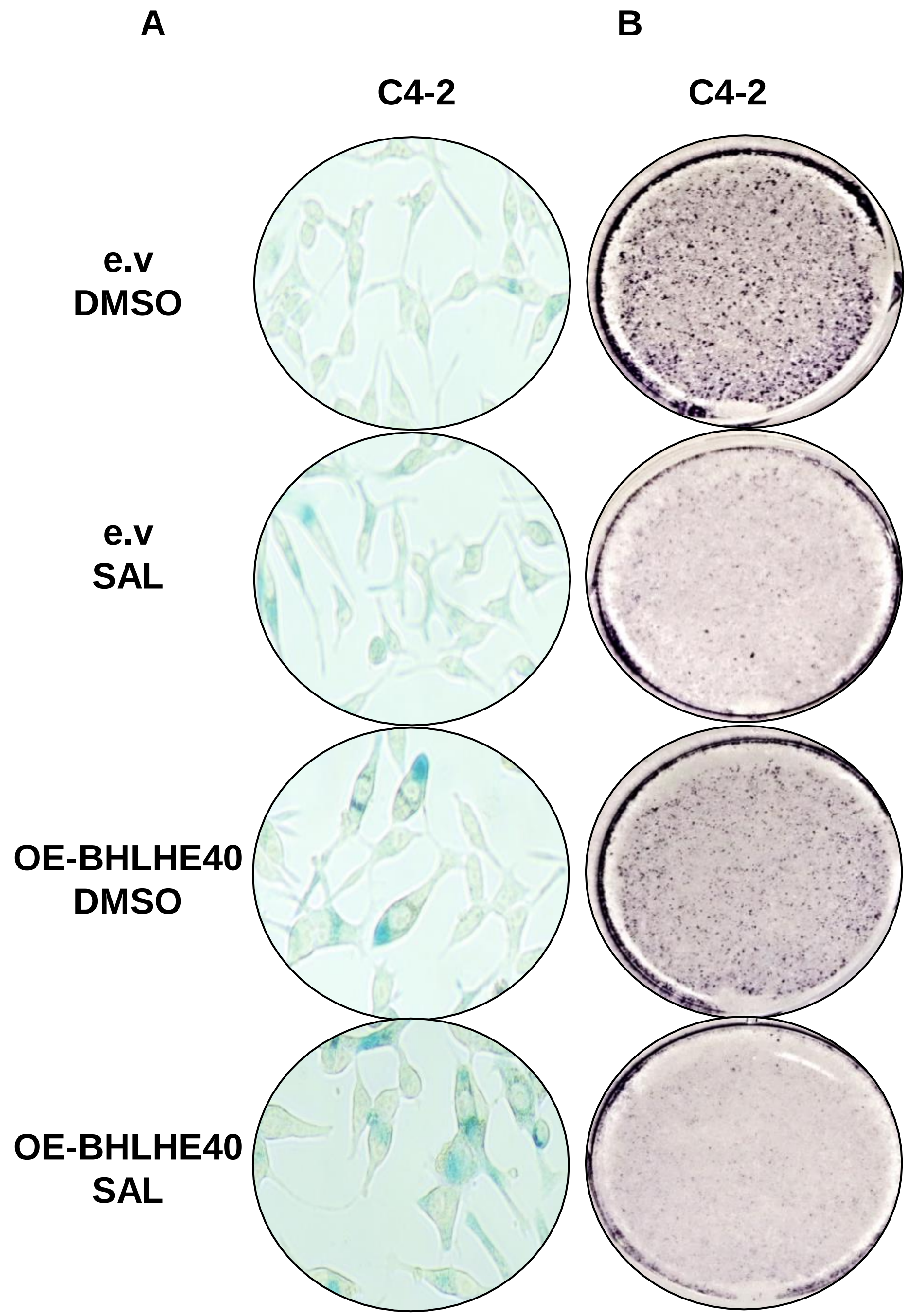

Fig. S9- Over-expression of BHLHE40 induces cellular senescence. A: Detection of SA β-Gal activity of treated cells for 72 h. **B:** Representative crystal violet staining showed reduction of growth by BHLHE40 over-expression.

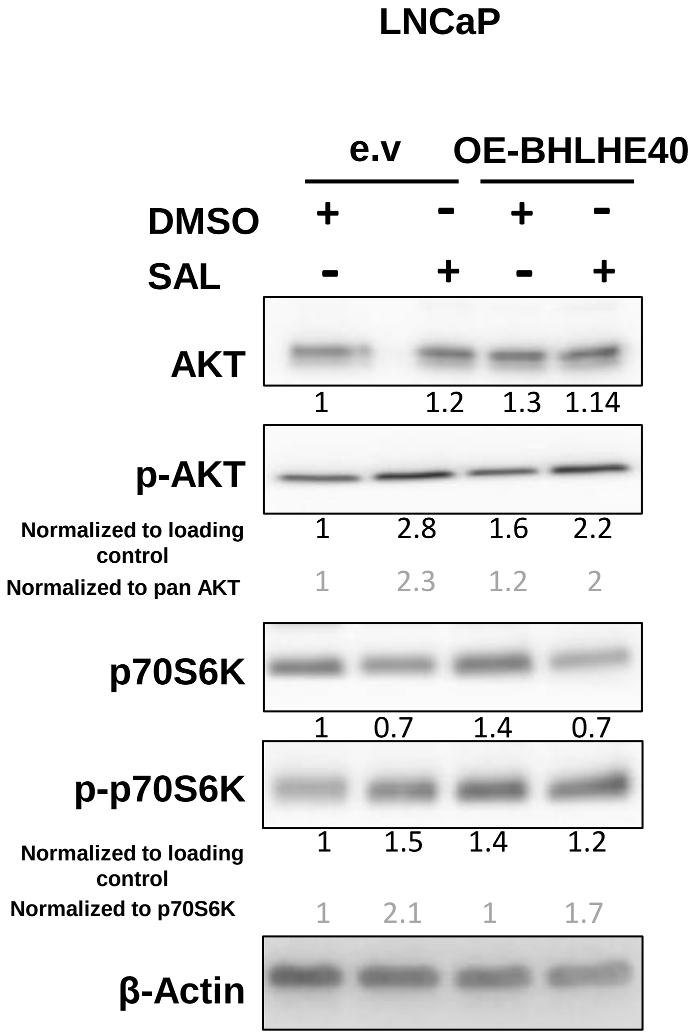

Fig. S10- BHLHE40 overexpression reduced the SAL-induced phosphorylation of AKT and p70S6K to basal level. Western blot data shows that phosphorylation of AKT and p70S6K were backed to basal level after overexpression of BHLHE40 in LNCaP cell line.

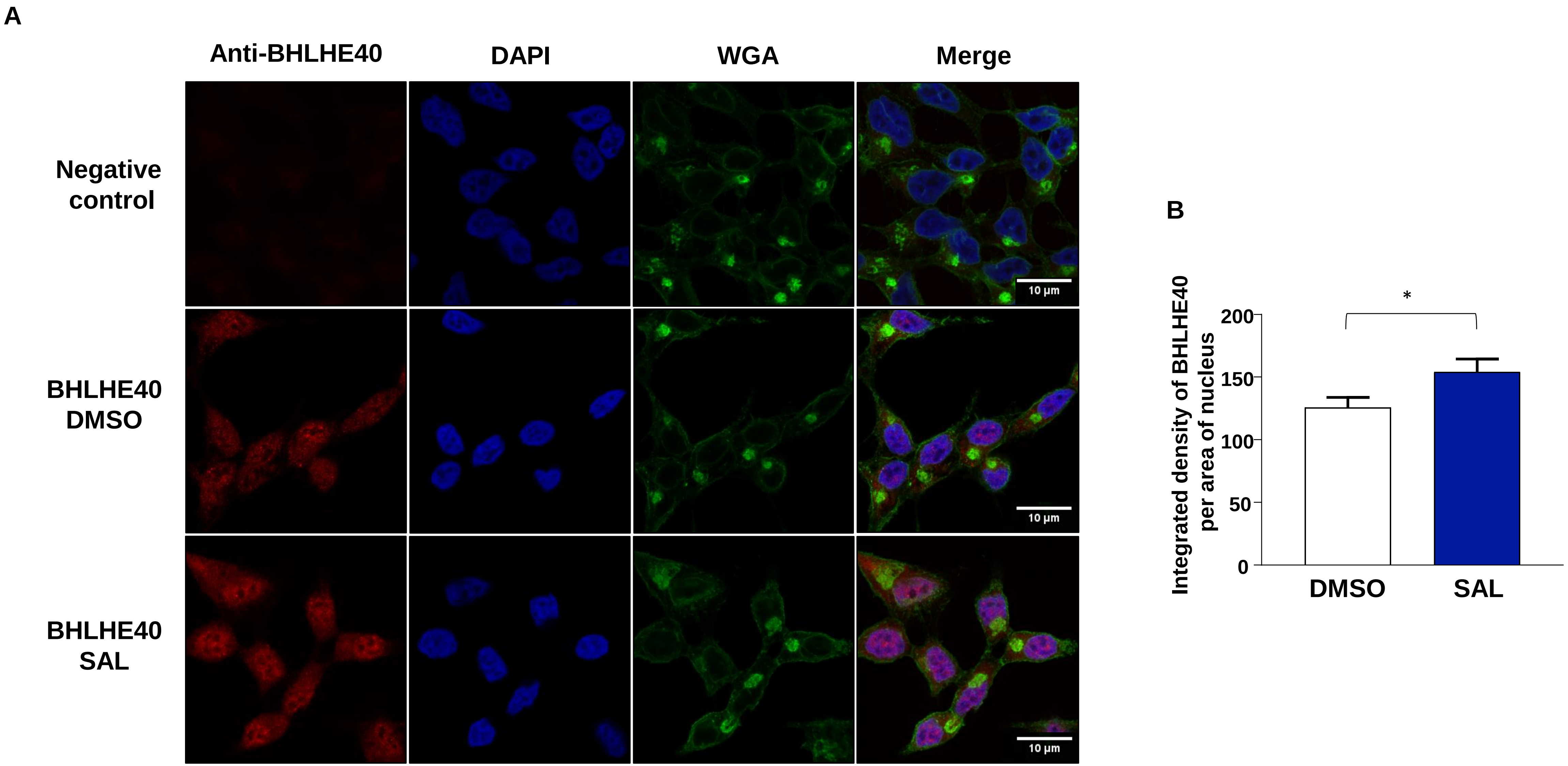

Fig. S11- SAL induced translocation of BHLHE40 to the nucleus in LNCaP cell line. A: Immunofluorescence staining was performed to detect the changes in the translocation of BHLHE40 after SAL treatment. SAL increase translocation of BHLHE40 to the nucleus in LNCaP cell line. **B:** Quantification of the integrated density of BHLHE40 in the nucleus.

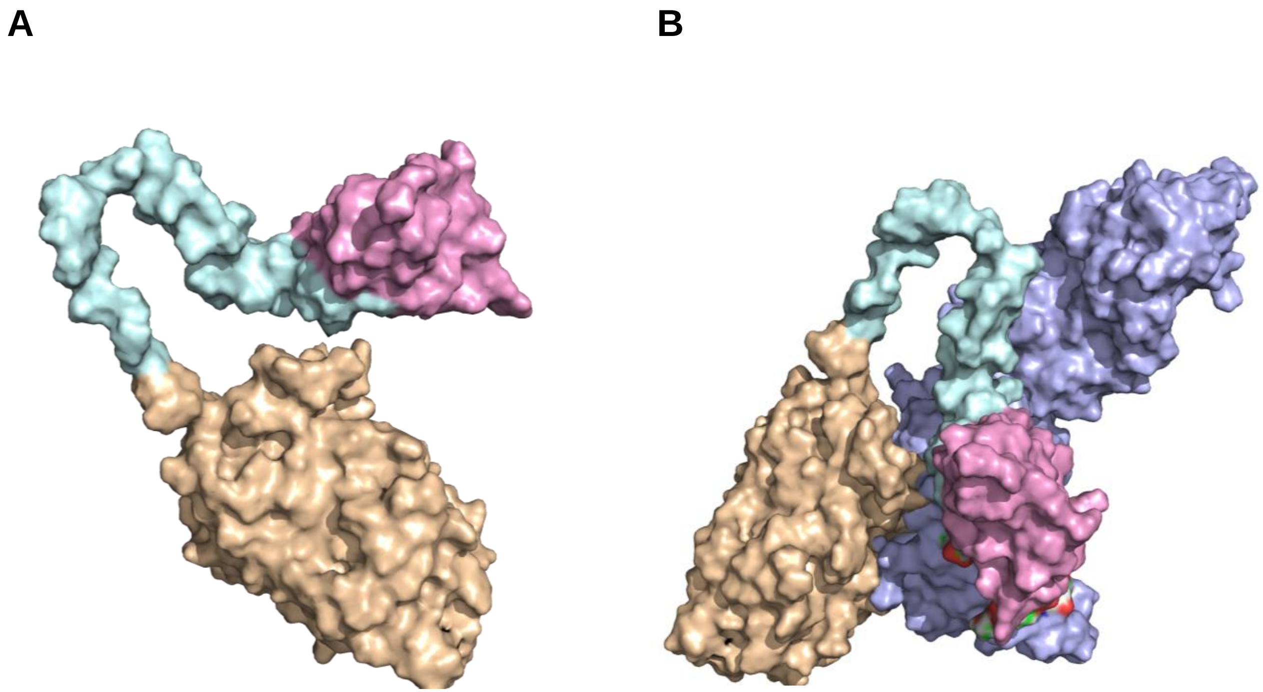

Fig. S12- Surface model of AR prediction and BHLHE40-AR interaction prediction. **A**: AR-DBD-hinge-LBD. **B**: Known crystal structure of AR-LBD with R1881 was used to predict binding of AR-DBD-LBD with BHLHE40 bound to DNA.
