# Supplementary material for: The clock gene BHLHE40 and atypical CCNG2 control androgen-induced cellular senescence as a novel tumor suppressive pathway in prostate cancer
Source: J Exp Clin Cancer Res. 2024 Jun 20;43:174. doi: 10.1186/s13046-024-03097-6 (PMC11188219; doi:10.1186/s13046-024-03097-6)
Supplement: Supplementary file 2 — Supplementary Material 2. [file 13046_2024_3097_MOESM2_ESM.docx]

**Supplementary tables:**

| Supplemental Table S1- Sequence of primers used in qRT-PCR (5’ ... 3’) | |
| --- | --- |
| BHLHE40 (DEC1) | FRW: ACTTACCTTGAAGCATGTGAAAGCA  REV: CATGTCTGGAAACCTGAGCAGAA |
| BHLHE41 (DEC2) | FRW: CTGATGCTGTTGCTCGGTTA  REV: TGACAACTCTGGGACATCTG |
| CDKN2B (p15) | FRW: GAATGCGCGAGGAGAACAAG  REV: TCATCATGACCTGGATCGCG |
| CCNG2 (Cyclin G2) | FRW: GTGTTCCTGAGCTGCCAACGAT  REV: AGGTGCACTCTTGATCACTGGG |
| KLK3 (PSA) | FRW: GAGGCTGGGAGTGCGAGAAG  REV: TTGTTCCTGATGCAGTGGGC |
| FKBP5 | FRW: GAGGAAACGCCGATGATTGGAGAC  REV: CATGCCTTGATGACTTGGCCTTTG |
| TMPRSS2 | FRW: CCTGCAAGGACATGGGCTATA  REV: CCGGCACTTGTGTTCAGTTTC |
| TBP | FRW: GATCTTTGCAGTGACCCAGCATCA  REV: CTCCAGCACACTCTTCTCAGC |
| TUBA (alpha-Tubulin) | FRW: TGGAACCCACAGTCATTGATGA  REV: TGATCTCCTTGCCAATGGTGTA |

| Supplemental Table S2- Antibodies | | | |
| --- | --- | --- | --- |
| Anti-Target | dilution | company | Cat No. |
| anti-BHLHE40 | 1:20000 for WB  1:500 for Co-IP | NOVUS Biological | NB100-1800 |
| anti-AR | 1:1000 for WB  1:500 for Co-IP | Merck Millipore | #06-680 |
| anti-p21WAF1/Cip1 | 1:1000 | Cell Signaling | #2946 |
| anti-p15INK4b | 1:2000 | MyBioSource | MBS821044 |
| anti-P70S6K | 1:1000 | Cell Signaling | #2708 |
| anti-p-P70S6K(T389) | 1:1000 | Cell Signaling | #9205 |
| anti-β-Actin | 1:10000 | Abcam | ab6276 |
| anti-c-PARP | 1:1000 | Cell Signaling | #9546 |
| anti-Pan-AKT | 1:1000 | Cell Signaling | #4685 |
| anti-p-AKT(S473) | 1:1000 | Cell Signaling | #4058 |
| anti-Ki67 | 1:200 | Abcam | ab243878 |
| anti-mouse IgG | 1:10000 | Cell Signaling | #7076S |
| anti-rabbit IgG | 1:10000 | Cell Signaling | #7074S |
| anti-rabbit IgG Alexa 546 | 1:1000 | Invitrogen | A11035 |

| Supplemental Table S3- Gleason score of prostatectomy samples | |
| --- | --- |
| Patient 1 | 7 |
| Patient 2 | 6 |
| Patient 3 | 7b |
| Patient 4 | 7a |
| Patient 5 | 7b |
| Patient 6 | 9 |

| Supplemental Table S4- pathways-log2FC of specific genes for BHLHE40 KD SAL vs. Control SAL | p-value |
| --- | --- |
| Circadian Clock | 0.007085 |
| Signaling by MET | 0.010856 |
| Transcriptional Regulation by TP53 | 0.004968 |
| Cellular response to hypoxia | 0.000188 |
| VEGFA-VEGFR2 Pathway | 0.000134 |
| Chaperonin-mediated protein folding | 0.000242 |
| RUNX3 regulates NOTCH signaling | 0.046931 |
| Translation | 4.51E-05 |

| Supplemental Table S5- Pathways-genes containing AR and bHLH motifs from AR C4-2 ChIP-seq | p-value |
| --- | --- |
| FOXO-mediated transcription | 1.09E-10 |
| Circadian Clock | 3.77E-09 |
| Heme signaling | 5.88E-08 |
| Cellular Senescence | 3.44E-07 |
| FOXO-mediated transcription of cell cycle genes | 7.63E-06 |
| BMAL1: CLOCK, NPAS2 activates circadian gene expression | 5.70E-05 |
| Signaling by Hedgehog | 0.021495 |
